# Genome-wide analysis of blood lipid metabolites in over 5000 South Asians reveals biological insights at cardiometabolic disease loci

**DOI:** 10.1186/s12916-021-02087-1

**Published:** 2021-09-10

**Authors:** Eric L. Harshfield, Eric B. Fauman, David Stacey, Dirk S. Paul, Daniel Ziemek, Rachel M. Y. Ong, John Danesh, Adam S. Butterworth, Asif Rasheed, Taniya Sattar, Zameer-ul-Asar, Imran Saleem, Zoubia Hina, Unzila Ishtiaq, Nadeem Qamar, Nadeem Hayat Mallick, Zia Yaqub, Tahir Saghir, Syed Nadeem Hasan Rizvi, Anis Memon, Mohammad Ishaq, Syed Zahed Rasheed, Fazal-ur-Rehman Memon, Anjum Jalal, Shahid Abbas, Philippe Frossard, Danish Saleheen, Angela M. Wood, Julian L. Griffin, Albert Koulman

**Affiliations:** 1 British Heart Foundation Cardiovascular Epidemiology Unit, Department of Public Health and Primary Care, University of Cambridge, Cambridge, CB1 8RN UK; 2 Stroke Research Group, Department of Clinical Neurosciences, University of Cambridge, Cambridge, CB2 0QQ UK; 3 Internal Medicine Research Unit, Pfizer Worldwide Research, Development and Medical, Cambridge, Massachusetts 02139 USA; 4 British Heart Foundation Centre of Research Excellence, University of Cambridge, Cambridge, CB2 0QQ UK; 5 National Institute for Health Research Blood and Transplant Research Unit in Donor Health and Genomics, University of Cambridge, Cambridge, CB1 8RN UK; 6 National Institute for Health Research Cambridge Biomedical Research Centre, University of Cambridge and Cambridge University Hospitals, Cambridge, CB2 0QQ UK; 7 Health Data Research UK Cambridge, Wellcome Genome Campus and University of Cambridge, Cambridge, CB10 1SA UK; 8 Department of Human Genetics, Wellcome Sanger Institute, Hinxton, CB10 1SA UK; 9 Inflammation and Immunology, Pfizer Worldwide Research, Development and Medical, 10785 Berlin, Germany; 10 Center for Non-Communicable Diseases, Karachi, 75300 Pakistan; 11 National Institute of Cardiovascular Diseases, Karachi, 75510 Pakistan; 12 Punjab Institute of Cardiology, Lahore, 42000 Pakistan; 13 Karachi Institute of Heart Diseases, Karachi, 75950 Pakistan; 14 Red Crescent Institute of Cardiology, Hyderabad, 71500 Pakistan; 15 Faisalabad Institute of Cardiology, Faisalabad, 38000 Pakistan; 16 Department of Biostatistics & Epidemiology, University of Pennsylvania, Philadelphia, Pennsylvania 19104 USA; 17 Department of Biochemistry and Cambridge Systems Biology Centre, University of Cambridge, Cambridge, CB2 1GA UK; 18 Section of Biomolecular Medicine, Division of Systems Medicine, Department of Metabolism, Digestion, and Reproduction, Imperial College London, London, SW7 2AZ UK; 19 Core Metabolomics and Lipidomics Laboratory, National Institute for Health Research, Cambridge Biomedical Research Centre, Cambridge, CB2 0QQ UK

**Keywords:** Lipidomics, Genetics, Gaussian Graphical Modelling, Network analysis, South Asian

## Abstract

**Background:**

Genetic, lifestyle, and environmental factors can lead to perturbations in circulating lipid levels and increase the risk of cardiovascular and metabolic diseases. However, how changes in individual lipid species contribute to disease risk is often unclear. Moreover, little is known about the role of lipids on cardiovascular disease in Pakistan, a population historically underrepresented in cardiovascular studies.

**Methods:**

We characterised the genetic architecture of the human blood lipidome in 5662 hospital controls from the Pakistan Risk of Myocardial Infarction Study (PROMIS) and 13,814 healthy British blood donors from the INTERVAL study. We applied a candidate causal gene prioritisation tool to link the genetic variants associated with each lipid to the most likely causal genes, and Gaussian Graphical Modelling network analysis to identify and illustrate relationships between lipids and genetic loci.

**Results:**

We identified 253 genetic associations with 181 lipids measured using direct infusion high-resolution mass spectrometry in PROMIS, and 502 genetic associations with 244 lipids in INTERVAL. Our analyses revealed new biological insights at genetic loci associated with cardiometabolic diseases, including novel lipid associations at the *LPL*, *MBOAT7*, *LIPC*, *APOE-C1-C2-C4*, *SGPP1*, and *SPTLC3* loci.

**Conclusions:**

Our findings, generated using a distinctive lipidomics platform in an understudied South Asian population, strengthen and expand the knowledge base of the genetic determinants of lipids and their association with cardiometabolic disease-related loci.

**Supplementary Information:**

The online version contains supplementary material available at 10.1186/s12916-021-02087-1.

## Background

Mass spectrometry-based lipidomics, which aims to capture information on the full complement of lipid metabolites in a given biological sample [1], holds the potential to identify novel insights leading to lipid regulation and dyslipidaemia, potentially providing new mechanisms that link lipid perturbances with cardiometabolic disorders. While pathways underlying dyslipidaemia have been widely studied, we still do not understand how individual lipid species are regulated or contribute to disease. With increasing rates of cardiometabolic diseases in low- and middle-income countries, there is a need for well-powered studies to understand the mechanisms that lead to such disorders in these settings. This need is especially acute for genetic studies where the overrepresentation of individuals of European ancestry amongst genotyped cohorts has led to ancestral bias in effect size estimates at both the genotype and polygenic score levels [2].

In this study, we aimed to identify novel genetic associations with lipid metabolites in an understudied South Asian population and determine plausible metabolic pathways for the significantly associated lipid metabolites. We performed a comprehensive interrogation of genetic influences on the human blood serum lipidome using direct infusion high-resolution mass spectrometry (DIHRMS). We quantified 340 lipid metabolites in 5662 individuals from Pakistan, from which we identified 253 genotype–lipid associations (lipid quantitative trait loci, or lipid QTLs [3, 4]) at 24 independent loci, providing new insights into lipid metabolism and its impact on cardiovascular and metabolic diseases.

To help disentangle which of these findings are specific to the Pakistani population and which are unique to the lipid platform itself, we also carried out a parallel set of analyses using the same lipidomics platform in a much larger cohort of individuals from the UK. We measured 399 lipid metabolites in 13,814 healthy British blood donors, from which we identified 502 lipid QTLs at 38 independent loci.

## Methods

### Study descriptions

Our primary analyses involved a subset of participants from the Pakistan Risk of Myocardial Infarction Study (PROMIS), a case-control study of first-ever acute myocardial infarction (MI) in nine urban centres in Pakistan consisting of approximately 16,700 cases and 18,600 controls. Details of PROMIS have been described previously [5]. In this analysis, we analysed controls (individuals free from MI at baseline), who were identified and recruited at the same hospitals as cases according to the following order of priority: (1) visitors of patients attending the outpatient department, (2) patients attending outpatient clinics for non-cardiac-related symptoms, and (3) non-first-degree relative visitors of MI cases. The present analysis involved serum samples from 5662 PROMIS controls for which genetic and lipid-profiling data were available. Ethical approval was obtained from the relevant ethics committee of each of the institutions involved in participant recruitment and the Center for Non-Communicable Diseases in Karachi, Pakistan, and informed consent was obtained from each participant recruited into the study, including for use of samples in genetic, biochemical, and other analyses.

Comparative, parallel analyses were performed in INTERVAL, a prospective cohort study of approximately 50,000 healthy blood donors from the UK, the details of which have been described previously [6]. In this study, we analysed data from 13,814 participants with genetic and lipid-profiling data.

### Lipid profiling

Lipid levels in human serum were quantified using direct infusion high-resolution mass spectrometry (DIHRMS), as we described previously [7]. In brief, we extracted lipids from the serum samples using an Anachem Flexus automated liquid handler (Anachem, Milton Keynes, UK) and transferred the samples to 96-well plates using a 96-head microdispenser (Hydra Matrix, Thermo Fisher Ltd., Hemel Hampstead, UK). We then used a Triversa Nanomate (Advion, Ithaca, USA) to infuse the samples into an Exactive Orbitrap (Thermo, Hemel Hampstead, UK), which acquired the lipid signal data. Data processing, peak-picking, normalisation, cleaning, and quality control were performed to identify and record signals for 340 known lipids in 5662 PROMIS participants. The 340 lipids corresponded to five broad lipid categories (fatty acyls and derivatives, glycerolipids, glycerophospholipids, sphingolipids, and sterol lipids), which are further subdivided into fourteen lipid subclasses (Supplementary Table 3 in Additional file 2). Our peak-picking algorithm [7] selected all lipids within an *m/z* window of 185–1000, using a time window of 20–70 s for lipids in positive ionisation mode and 95–145 s for lipids in negative ionisation mode. A lipid list containing all known lipids within this *m/z* range was used to extract information on the lipid concentrations at specific peaks of interest, consisting of 1305 lipids in positive ionisation mode and 3772 lipids in negative ionisation mode. Quality control samples and blanks were used to remove lipids that were not able to be detected or had poor quality of assessment, resulting in a final list of 340 distinct lipid annotations across both ionisation modes. We normalised each lipid by expressing the intensity as a proportion of the total signal for each participant and then applied a log transformation to obtain an approximately normal distribution.

We performed DIHRMS on INTERVAL participants using the same protocol. We obtained data on 399 lipids, 228 in positive ionisation mode, and 171 in negative ionisation mode, which make up 19 lipid subclasses (Supplementary Table 1).

### Genotyping and imputation

DNA from PROMIS participants was extracted from leukocytes in Pakistan and genotyped at the Wellcome Sanger Institute in Cambridge, UK, on either (1) the Illumina 660-Quad GWAS platform, which consisted of 527,925 genotyped autosomal variants after quality control (QC) steps were performed, or (2) the Illumina HumanOmniExpress GWAS platform, which consisted of 643,333 genotyped autosomal variants after QC. Genetic samples were removed if (1) they were heterozygosity outliers (heterozygosity > mean ± 3 SD), (2) the sample call rate was less than 97%, (3) there was discordant sex between genetically inferred and self-reported sex, or (4) they were duplicate or related pairs (kinship coefficient > 0.375). Single nucleotide polymorphisms (SNPs) were excluded if (1) the SNP call rate was less than 97%, (2) there was evidence of departure from Hardy-Weinberg Equilibrium (HWE) at a *P* value of less than 1 x 10^-7^, or (3) the minor allele frequency (MAF) was less than 1%. Imputation was applied to the cleaned PROMIS datasets using the 1000 Genomes Project March 2012 (v3) release [8] as the reference panel. Imputation was conducted using IMPUTE v2.1.0 [9] using 5-Mb non-overlapping intervals for the whole genome. Once imputation had been performed for the samples on both genotyping platforms separately, there were over 7.2 million imputed SNPs available for analyses in either dataset before further QC. SNPs were removed if they were poorly imputed, i.e., if they had an information score (an assessment of the level of accuracy of imputation) < 80%. The results were then extracted from the output files, and once the final QC filters were reapplied, 6,720,657 SNPs were available for analyses of the lipidomics data. In total, 5662 individuals from PROMIS had concomitant information on lipidomics data and imputed SNPs.

DNA from INTERVAL participants was extracted from buffy coat at LGC Genomics (UK) using a Kleargene method, and samples of sufficient concentration and purity were aliquoted for shipment to Affymetrix for genotyping [10]. Duplicate samples and samples that were not of European ancestry were excluded. Additionally, SNPs were excluded if (1) the variant had fewer than 10 called minor allele homozygotes, (2) the cluster plot contained at least one sample with an intensity at least twice as far from the origin as the next most extreme sample, (3) the outlying sample(s) had an extreme polar angle (<15° or >75°) in the direction of the minor allele, (4) call rate < 99% per batch and < 75% overall, (5) MAF < 0.05, (6) HWE *P* < 1 x 10^-6^, or (7) *r*^2^ ≥ 0.2 between pairs of variants [10]. The dataset was phased using SHAPEIT3 (in chunks of 5000 variants with an overlap of 250 variants between chunks) and subsequently imputed using the 1000 Genomes Phase 3-UK10K imputation panel, resulting in 87,696,910 imputed variants in the dataset [10]. In total, 13,814 individuals from INTERVAL had overlapping information on lipidomics data and imputed SNPs.

### Primary genome-wide association analyses

In PROMIS, linear regression was used to determine the association of each lipid with each SNP using SNPTEST v2.4.1 [11], which was performed separately for the samples genotyped on each of the two genetic platforms. Residuals were calculated from the null model for each lipid, which included adjustment for age group, sex, date of survey, plate (batch), and fasting status. To account for population stratification and genetic substructure in the data, principal component analysis was conducted on the multi-dimensional scaling matrix created from autosomal SNPs as implemented in PLINK; the first six principal components were subsequently added to each model. A missing data likelihood score test was used when testing for association at imputed SNPs to account for genotype uncertainty. Beta estimates and standard errors from the association results for the two genetic platforms were combined in a fixed-effect inverse-variance-weighted meta-analysis using METAL version 2011-03-25 [12]. The threshold for genome-wide significance level was set to *P* < 8.929 x 10^-10^, which corrected for multiple testing by dividing the standard genome-wide significance level (5 x 10^-8^) by the number of principal components (56) that explained over 95% of the variance in the levels of the lipids. All traits gave genomic inflation factors (*λ*) in the meta-analysis less than 1.05 [mean (SD) 1.0139 (0.0129); range 0.9741–1.0455], indicating that there was little evidence of systematic bias in the test statistics.

To verify the robustness and validity of the results, post-analysis quality control (QC) was performed by comparing the meta-analysis results with the results on each GWAS platform. The lead SNPs from the meta-analysis were only kept if they (1) passed QC in the raw SNPTEST results from both GWAS platforms (i.e. HWE *P* < 1 x 10^-7^, call rate < 0.97, MAF < 0.01, and info score < 0.80); (2) had beta (*β*) estimates in the same direction on both platforms (i.e. betas were both negative or both positive); and (3) had *P* < 0.01 on both platforms (with *P* < 8.9 x 10^-10^ in the meta-analysis).

In INTERVAL, linear regression was performed using BOLT-LMM v2.2 [13] to determine the association of each lipid with each SNP. Residuals were calculated from the null model for each lipid with adjustment for plate, age, sex, centre, appointment month, appointment hour, processing time in hours, and the first three genetic principal components. The threshold for genome-wide significance level was set to *P* < 4.464 x 10^-10^ (5 x 10^-8^/112), as 112 principal components explained >95% of the variance in lipid levels.

### Genome-wide analysis of ratios of lipids

A second discovery step was carried out in PROMIS by testing genome-wide associations on 26 pairwise ratios of lipid concentrations. Ratios were identified based on those that had strong biological rationales and that acted through thoroughly understood metabolic pathways (list of ratios with rationales and references provided in Supplementary Table 7). Meta-analysis was performed to combine results from the two genotyping platforms using a fixed-effect inverse-variance weighted meta-analysis. Since there were fewer statistical tests for the ratios than for the individual lipids, the combined result file for each ratio was filtered using the standard threshold for genome-wide significance of *P* < 5 x 10^-8^.

### Conditional analyses

We conducted conditional analyses on the significant loci from the meta-analysis results of the univariate GWAS for each lipid in PROMIS. All SNPs were selected where *P* < 8.9 x 10^-10^, the 5-Mb chunks were identified where each of these SNPs were located, and the lead SNPs were selected within each chunk that had the strongest *P* value. On an individual lipid basis, for each 5-Mb chunk that was identified, SNPTEST was run on the imputed data for each genotyping platform using the same null model as before, except also conditioning on the lead SNP in the identified chunk. The results from the samples analysed on each genotyping platform were combined in a meta-analysis using METAL as described above, and any SNPs where *P* < 8.9 x 10^-10^ were identified. The lead SNP from the meta-analysed results of the first conditional analysis (i.e. the SNP with the strongest *P* value) was identified, and this process was repeated for each chunk. Additional SNPs to be conditioned on were repeatedly added to the model on each chunk for each lipid until there were no more significant SNPs left within that chunk. The final set of SNPs that were “conditionally independent” for each lipid were combined into a single list across all lipids, resulting in 253 SNP-lipid associations (lipid QTLs) for 181 lipids, or 90 unique lead SNPs. These variants were grouped into 24 loci using a distance measure of ±500 Kb.

We identified the proportion of variation in the lipidome explained by inherited genetic variants by regressing each lipid on the number of copies of each allele held by each participant for each of the conditional analysis sentinel SNPs.

As a sensitivity analysis, we also conducted a GWAS for all significant lipids from the conditional analyses using the same regression models with additional adjustment for several clinical lipid measures (total cholesterol, HDL-C, and triglycerides).

### Candidate gene annotation

In order to prioritise candidate genes that might underpin the genotype—lipid associations, we applied the ProGeM framework (Supplementary Figure 6 in Additional file 1) to both PROMIS and INTERVAL [14]. In addition to reporting the nearest gene to the sentinel variant, ProGeM combines information from complementary “bottom-up” and “top-down” approaches to assess the credibility of potential candidate genes [14] (Supplementary Table 9).

For the bottom-up approach, we annotated SNPs based on their putative effects on proximal gene function if any of the following conditions were met: (1) the SNP resided within an exonic sequence of a gene (Supplementary Table 9), (2) the SNP resided within a splice-site (±2 bp from an intron-exon boundary), (3) the SNP was in high linkage disequilibrium (LD) (*r*^2^ ≥ 0.8) with a non-synonymous SNP (Supplementary Table 10), and/or (4) the SNP was a *cis*-eQTL for a local gene (Supplementary Table 11). To identify any exonic and splice site SNPs within our SNP list, we ran the Variant Effect Predictor (VEP) (http://www.ensembl.org/common/Tools/VEP?db=core) on the list of variants with the “pick” option (which outputs one block of annotation per variant) and used Ensembl transcripts as the reference for determining consequences. SNPs in high LD with our list of associated SNPs were identified within our imputed dataset and run through VEP to select only non-synonymous SNPs. *Cis*-eQTLs within our list of associated SNPs were identified using eQTL data provided by the Genotype-Tissue Expression (GTEx) project (http://www.gtexportal.org/home/datasets), keeping only significant SNP-gene associations (filename: “GTEx_Analysis_v7_eQTL.tar.gz”). We only annotated SNPs if they were significant eQTLs in at least one of the following tissues we deemed most relevant for lipid-related phenotypes: subcutaneous adipose tissue, visceral adipose tissue, liver, and/or whole blood.

In the top-down approach, for each of our associated SNPs, we first identified all proximal genes located ≤ 500-Kb upstream or downstream using the ANNOVAR tool (http://annovar.openbioinformatics.org/en/latest/). We then identified all genes previously associated with a lipid-related biological process or function from the following databases: (1) LIPID MAPS Proteome Database (LMPD) (http://www.lipidmaps.org/data/proteome/LMPD.php), (2) Gene Ontology (GO) (http://geneontology.org/), (3) Online Mendelian Inheritance in Man catalogue (OMIM), (4) Mouse Genome Informatics (MGI) database (http://www.informatics.jax.org/), (5) Kyoto Encyclopedia of Genes and Genomes (KEGG) (http://www.genome.jp/kegg/), and/or (6) Ingenuity Pathway Analysis (IPA) (http://www.ingenuity.com/products/ipa).

LMPD is an object-relational database of lipid-associated genes and proteins across multiple species including human, mouse, and fruit fly; we simply extracted all human genes (1116 genes in total) from this database (accessed 16-Mar-2016). For GO and OMIM, we first identified all terms or Mendelian diseases containing one or more lipid-related keyword(s) using HumanMine (http://www.humanmine.org/), then we extracted all human genes associated with one or more of these terms (accessed 01-Apr-2016 and 07-Apr-2016). Similarly, for MGI we extracted all mouse genes using MouseMine (http://www.mousemine.org/mousemine/begin.do) (accessed 31-Mar-2016) that were associated with the following manually selected lipid-related terms and their children: (1) abnormal lipid homeostasis (MP:0002118), (2) abnormal lipoprotein level (MP:0010329), (3) abnormal lipid metabolism (MP:0013245), and (4) adipose tissue phenotype (MP:0005375). From the KEGG database, we extracted all lipid compounds (with “C” number IDs) with biological roles in order to identify all genes associated with reactions (with “R” number IDs) involved in lipid biology using MitoMiner (http://mitominer.mrc-mbu.cam.ac.uk/release-3.1/begin.do) (accessed 31-Mar-2016). Finally, from IPA, we downloaded the interaction networks for all fourteen of the lipid subclasses in order to extract all genes in a compound-specific manner (accessed 13-Apr-2016).

Once we had obtained lists of lipid-related genes from the aforementioned databases, we then searched for overlap with our list of proximal (≤ 500 Kb) genes based on HUGO Gene Nomenclature Committee (HGNC) symbols, thereby annotating SNPs with proximal genes where there was evidence that at least one might be involved in lipid-related biology. For each lead SNP, we first recorded whether there was any compound-specific evidence from IPA for a SNP-gene assignment whereby both the SNP (from this study) and the gene (from IPA) were associated with the same lipid subclass. Then, from the five remaining (compound non-specific) databases, we categorised overlapping genes as either (1) recurrent candidates, in that they were highlighted in at least two different databases, or (2) single candidates. Further, we assigned the recurrent candidates a score out of five for prioritisation purposes, with one point awarded for each database highlighting them as being lipid-related.

After performing comprehensive annotation of SNPs as per the bottom-up and top-down procedures, we then integrated this information to try to predict the most likely causal gene(s) using a hierarchical approach as follows: (1) For those lead SNPs where the same gene was highlighted by both the bottom-up and the top-down approach, we selected this gene as the putative causal gene; (2) If both the SNP (from this study) and the proximal gene (from IPA) were associated with the same lipid subclass, we made further SNP-gene assignments accordingly; (3) finally, for each of the remaining lead SNPs, we assigned the highest scoring top-down gene and any bottom-up genes as the likely causal gene(s).

Separately, we assigned an expertly curated causal gene to each variant and compared the predicted causal genes identified by the functional annotation pipeline to assess concordance and validate the pipeline.

### Gaussian Graphical Modelling

As described previously [7], we estimated a Gaussian Graphical Model (GGM) on the normalised relative intensities of the lipids in PROMIS to better resolve lipid cross-correlations. The GGM resulted in a set of edges in which each edge connected two detected lipids if their cross-correlation conditioned on all other lipids was significantly different from zero. Subjects with more than 10% missing lipids as well as lipids with more than 20% missing subjects were removed from the analysis. The “genenet” R package was used to infer the GGM [15]. A similar approach for metabolomics data has been suggested previously [16]. To focus on strong effects, we retained only edges in the model that met an FDR cutoff of 0.05 and had a partial correlation coefficient greater than 0.2.

### Fatty acid chain enrichment analysis

We manually annotated detected lipids in PROMIS with their constituent fatty acid chains. For each combination of fatty acid chains, we counted the number of GGM edges connecting lipids with that specific combination, which we used to directly estimate *P* values of enrichment and depletion. To test whether edges from the GGM were enriched for any combination of fatty acid chains, we permuted the annotation 1000 times using the R package “BiRewire” [17], keeping the number of annotations per lipid and fatty acid chain constant.

### Network of genetic and metabolic associations

We used Cytoscape v3.2.1 [18] to generate a network of associations between genes and lipid subclasses in PROMIS (Fig. 3). Using a previously described approach [19], we constructed a GGM to connect lipids to each other based on partial correlation coefficients, and we also connected lipids with genetic loci using the conditional analysis results, with one link for each genome-wide significant association. The full network facilitates visualisation of the genetic determinants of human metabolism and the relationships between genetic loci and lipid subclasses.

The network diagrams were created by combining two parts to integrate different sources of information. The first part was created by loading the reported associations between lipids and genes into Cytoscape. Lipid species were clustered according to the lipid subclass they belong to, resulting in fourteen distinct lipid subclass nodes in the network. The 90 identified lead SNPs from the conditional analyses were clustered according to their corresponding predicted causal gene(s), which was determined using the ProGeM framework [14]. In cases where it was not possible to confidently identify a single predicted causal gene, loci were entered into the network instead. For the second part, a functional interaction network consisting solely of our list of predicted causal genes/loci was created in Cytoscape using interaction network data downloaded from IPA that had been merged using in-house R scripts to create a .sif file. For loci with multiple potential causal genes, interaction networks for all genes were extracted from IPA and an edge was drawn if at least one gene at that locus functionally interacts with another of our lipid-associated genes according to IPA. Finally, these two parts were merged together by node names (i.e. gene symbols). No enrichment statistics (e.g. KEGG pathways or GO terms) or other statistical information was used to produce the network, since this information was already incorporated to inform the predictions of the most likely “causal” genes, and would therefore invalidate the conclusions if it was also used to inform the network.

A second network diagram was created containing a subset of the first network containing only the triglyceride species (Fig. 4). It also provides more detail as it shows the individual triglycerides rather than the lipid subclass as a whole. Thus, it portrays the partial correlations of the triglycerides with each other and the association of each triglyceride with genetic loci.

## Results

### Genetic architecture of the lipidome in South Asians and in the UK

**Fig. 1:**
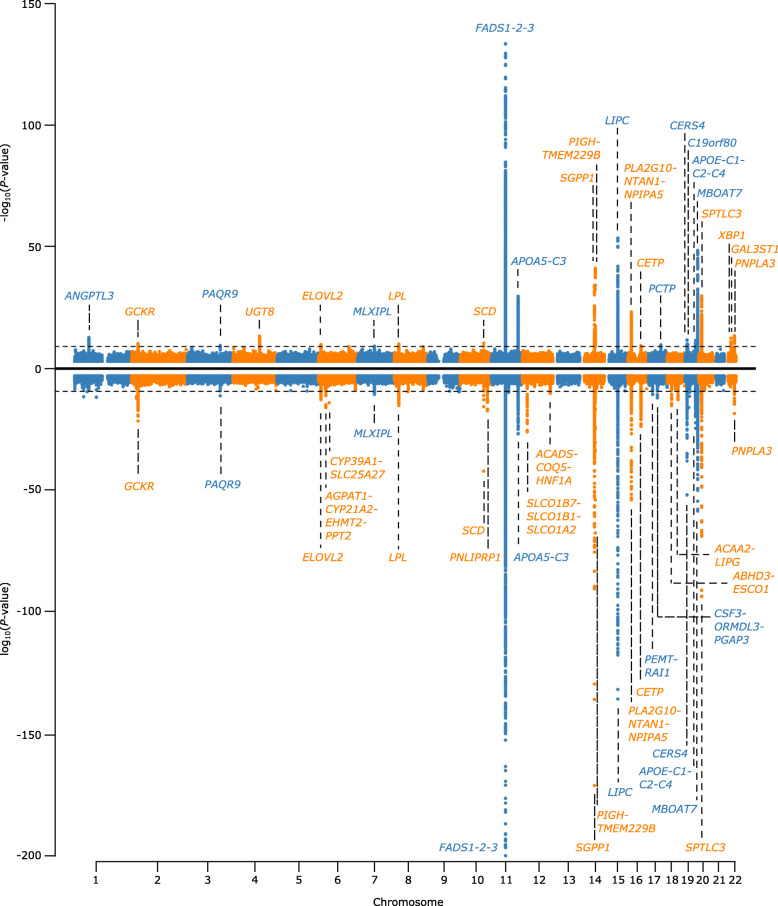
Miami plot of combined association results from genome-wide association analysis for all lipids in PROMIS and INTERVAL. The combined association results are shown for all lipids with each variant in PROMIS (top) and INTERVAL (bottom). *P* values > 1 x 10^-3^ have been truncated at 1 x 10^-3^, and *P* values < 1 x 10^-200^ have been truncated at 1 x 10^-200^. Actual *P* value for lead SNP in *FADS-1-2-3* locus in INTERVAL is 1.6 x 10^-286^

**Fig. 2:**
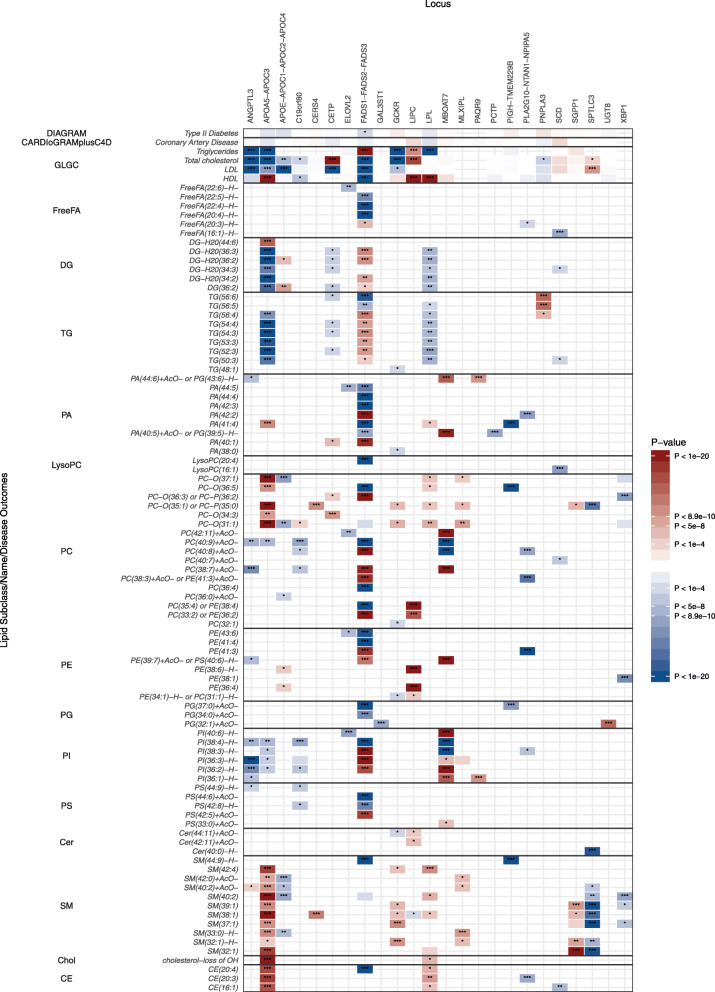
Heat map showing associations of significant loci from conditional analyses with selected lipid metabolites in PROMIS. The effect estimates of the associations between significant variants and selected lipids are plotted as a heat map. Results are shown for selected top lipids with the strongest associations within each subclass (rows) against the most strongly associated genetic variant within each locus (columns). The associations with major lipids from the GLGC (total cholesterol, HDL-C, LDL-C, and triglycerides), DIAGRAM Consortium (type 2 diabetes), and CARDIoGRAMplusC4D Consortium (coronary artery disease) are also shown. The magnitude and direction of the effect estimates (standardised per 1-SD) are indicated by a colour scale, with blue indicating a negative association and red indicating a positive association with respect to the SNP effect on the trait. Asterisks indicate the degree of significance of the *P* values of association. * = *P* < 1 x 10^-4^; ** = *P* < 5 x 10^-8^; *** = *P* < 8.9 x 10^-10^

We performed a genome-wide association study (GWAS) on the levels of 340 lipid metabolites using 6.7 million imputed autosomal variants in 5662 hospital-based controls from PROMIS. We applied DIHRMS to quantify serum lipid metabolites across five broad lipid categories, i.e. fatty acyls and derivatives, glycerolipids, glycerophospholipids, sphingolipids, and sterol lipids [7]. We demonstrated the robustness of these lipid measurements in several ways, including validation of lipid signals against blanks, pooled samples, and internal standards, with a median coefficient of variation of 11.60% (range 5.4–51.9), as we described previously [7]. Additionally, we replicated known associations of lipid metabolites with previously reported major lipid loci (Supplementary Table 17). After Bonferroni correction for multiple testing of variants and lipid metabolites (*P* < 8.929 × 10^-10^), we found 253 significant associations between 181 lipid metabolites and 24 genomic regions (Figs. 1 and 2, Supplementary Figure 1, Supplementary Table 4). The majority of these lipid metabolites (67%; *n* = 171) were associated with variation at a single locus, while 26% of lipid metabolites were associated with two loci and 7% were associated with three or more loci (Supplementary Figures 2a and 3). To detect multiple independent associations at the same locus, we used stepwise conditional analysis, identifying 90 conditionally independent variants associated with lipid metabolites (Supplementary Table 5). 335 (93%) of the lipid QTLs had multiple conditionally significant associations (Supplementary Figure 2b).

Using the same DIHRMS platform, we also performed a GWAS on levels of 399 lipid metabolites using 87.7 million imputed autosomal variants in 13,814 British blood donors from INTERVAL. We identified significant associations with lipids at 38 independent loci (Fig. 1, Supplementary Table 6). There was considerable consistency in the genomic regions identified in each study, with 18 (75%) of the significant genetic loci from PROMIS also found in INTERVAL (Fig. 1). Six genetic loci were specific to lipid levels in the Pakastani population: *ANGPTL3*, *UGT8*, *PCTP*, *C19orf80*, *XBP1*, and *GAL3ST1*. There were also twenty genetic loci associated with lipids in the British population that were not significantly associated with lipids in the Pakistani population. The beta estimates were consistent in magnitude and direction in both studies (Pearson correlation *r*=0.92), and the *P* values also showed consistency between studies (Supplementary Figure 3).

In PROMIS, the median proportion of variation in the lipidome explained by the genome-wide significant conditionally independent variants was 1.7% (interquartile range 1.5–1.9%) (Supplementary Figure 2c), which is slightly less than that reported in metabolomics studies [19–22] but similar to the reported variation explained in previous lipidomics studies [23, 24]. There was a strong inverse relationship between effect size and minor allele frequency (MAF) (Supplementary Figure 2d), consistent with previous GWAS of quantitative traits [10, 25]. Approximately 70% of the analysed genetic variants in this analysis were common (MAF >5%) and 30% were low-frequency (MAF: 1-5%) with a median MAF of 8%. To help identify candidate causal genes through which genetic loci may influence lipid levels and thereby impact disease risk, we applied the ProGeM framework [14] (Supplementary Tables 9-15, Supplementary Figure 6). We identified a plausible or established link to biochemical function for 16 of the 24 loci (including *GCKR*, *LPL*, *FADS1-2-3*, and *APOA5-C3*), involving 34 unique genes. In cases where it was not possible to annotate SNPs using our systematic approach, we assigned them to their nearest protein-coding gene.

Previous studies have shown that the ratios of metabolites can strengthen association signals and lead to a better understanding of possible mechanisms [19]. Thus, in addition to the individual lipid metabolites, we selected twenty-six ratios of lipid metabolites that act through well-understood metabolic pathways. These included ratios associated with lipase activity, elongases, docosahexaenoic acid (DHA) levels, dairy fat intake, insulin production, glucose control, de novo lipogenesis, and cardiovascular disease risk (Supplementary Table 7). Genome-wide association analyses of these ratios in PROMIS resulted in the identification of four additional loci that were not detected in the GWAS of individual lipid metabolites (*MYCL1-MFSD2A*, *LPGAT1*, *LOC100507470*, and *HAPLN4-TM6SF1*) (Supplementary Table 8).

Since most of the lipid species that we measured are present in lipoprotein particles, we explored whether the variance in clinical lipid measures (total cholesterol, HDL, and triglycerides) are likely to be major drivers of variation in lipid levels. Adjustment for clinical lipid measures showed that many associations were independent of the genetic variant’s effect on clinical lipids, including those in the *CERS4*, *CET4*, *ELOVL2*, *SCD*, and *UGT8* loci, and for lysophosphatidylcholines (Supplementary Table 18, Supplementary Figure 8). However, several associations (e.g. in the *FADS1-2-3*, *MBOAT7*, and *LIPC* loci, and for phosphatidylcholines, phosphatidylethanolamines, and sphingomyelins) attenuated substantially upon adjustment, suggesting that the genetic variants’ effects on specific lipid species are driven by their effect on clinical lipid measures.

### Network of genetic and metabolic associations

**Fig. 3:**
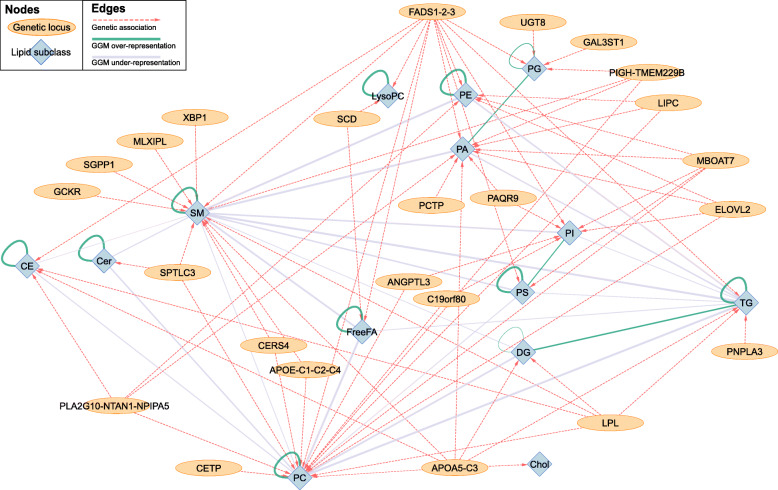
Combined network graph summarising genetic associations and a Gaussian graphical model (GGM) relating to levels of individual lipid species in PROMIS. Nodes representing genetic loci are each labelled with the most likely “causal” gene at that locus according to our functional annotation (see “Methods” section). In order for an edge to be drawn between a genetic locus and a lipid subclass, there must have been a minimum of one variant at that locus significantly (*P* < 8.9 x 10^-10^) associated with a minimum of one lipid species belonging to that lipid subclass. Edges between lipid subclasses indicate whether there was either a significant over- (green) or under- (purple) representation (the magnitude is indicated in the thickness of the edges) of GGM connections between lipid species belonging to different lipid subclasses

To identify and visualise the connectivity between lipid subclasses, we generated a network of genetic and metabolic associations in PROMIS by summarising within each subclass the pairwise partial correlations between lipid metabolites and their genetic associations (Fig. 3). This network diagram highlights that the number of connections between diglycerides and triglycerides was strongly over-represented in the Gaussian Graphical Model (GGM), indicating that there were more significant partial correlations between lipids from these subclasses than would be expected due to chance alone, whereas the number of connections between sphingomyelins and triglycerides was strongly under-represented in the GGM. In addition to being associated with variants from the *SPTLC3* and *FADS1-2-*3 loci, we found that sphingomyelins were associated exclusively with four loci that were not associated with any other lipid subclasses: *GCKR*, *SGPP1*, *MLXIPL*, and *XBP1*.

**Fig. 4:**
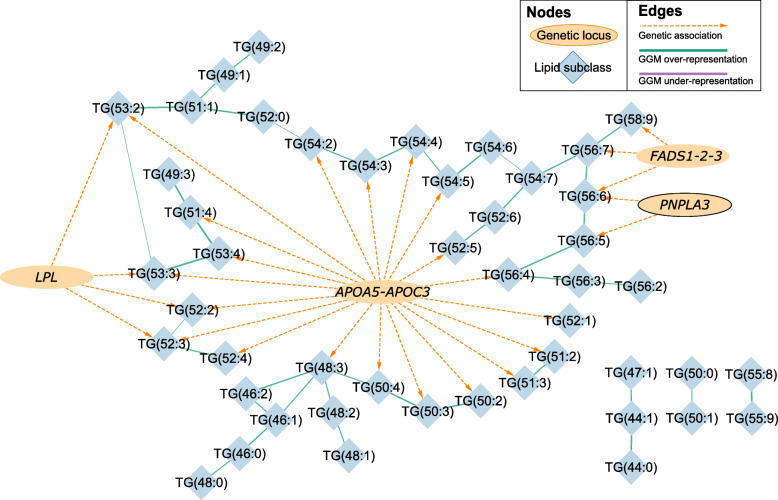
Combined network graph summarising genetic associations and a Gaussian graphical model (GGM) relating to levels of individual triglycerides in PROMIS. Nodes representing genetic loci are each labelled with the most likely “causal” gene at that locus according to our functional annotation (see “Methods” section). In order for an edge to be drawn between a genetic locus and a triglyceride, there must have been a minimum of one variant at that locus significantly (*P* < 8.9 x 10^-10^) associated with at least one triglyceride. Edges between triglycerides indicate whether there was either a significant over- (green) or under- (purple) representation, with the magnitude indicated by the thickness of the edges

Given the striking findings for triglycerides in the overall network diagram, we also generated a network in PROMIS for a subset of the triglyceride species showing the partial correlations of individual triglycerides and their detailed associations with genetic loci (Fig. 4). This network diagram shows that variants in the *APOA5-C3* locus are associated with a wide range of triglycerides, consistent with previous associations of Apolipoprotein A-V (ApoA5) with plasma triglyceride levels. ApoA5 is a component of a number of lipoprotein fractions including HDL, VLDL, and chylomicrons, and it may regulate the catabolism of triglyceride-rich lipoprotein particles by *LPL* and/or play a role in the assembly of VLDL particles [26–30]. The network mainly shows links with triglycerides containing polyunsaturated fatty acids (PUFAs), suggesting that variants in the *APOA5-C3* locus mainly affect the catabolism of lipoproteins containing triglycerides derived from adipose tissues that are relatively enriched in more unsaturated fatty acids. In contrast, we did not see direct links of fully saturated triglycerides with the *APOA5-C3* locus, suggesting that genetic variation at this locus is not particularly involved in the assembly of VLDL particles in the liver as part of de novo lipogenesis, in concordance with previous studies [31, 32] (see Supplementary Figure 5).

**Fig. 5:**
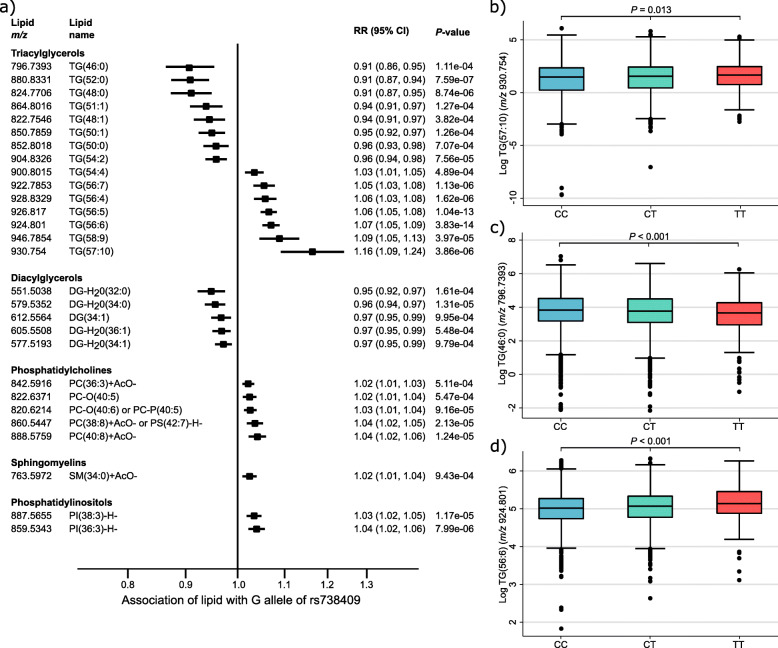
Association of lipids in PROMIS with *PNPLA3* and differences in levels of triglycerides by genotype. **a** Association of G allele of rs738409 in *PNPLA3* locus with levels of various lipids in PROMIS. The black lines denote 95% confidence intervals. Difference in levels of triglycerides in PROMIS by genotype: **b** [TG(57:10)+NH_4_]^+^ (*m/z* 930.754), **c** [TG(46:0)+NH_4_]^+^ (*m/z* 796.7393), and **d** [TG(56:6)+NH_4_]^+^ (*m/z* 924.801). *P* values are for ANOVA test of difference in mean levels of triglycerides by genotype

Fatty acid desaturase is key in the production of PUFAs; therefore, differences in *FADS1-2-3* activity are expected to be observed in triglycerides with a large number of double bonds and carbon atoms. Indeed, the GGM concords with established biochemistry since this locus is associated with triglycerides (TG) 56:6, 56:7, and 58:9 but is not associated with triglycerides with fewer double bonds or carbon atoms. In contrast, it is unclear why variants in the *PNPLA3* locus also have the strongest associations with triglycerides with a relatively larger number of carbon atoms and double bonds, namely TG(56:5) and TG(56:6) (see also Fig. 5). One possible explanation is that significantly associated variants in the *PNPLA3* locus are changing the substrate specificity so that there is a shift in the relative amounts of triglycerides that are exported from the liver.

Additionally, the network diagram confirms that *LPL* is mainly active on MUFAs in triglyceride species. Variants in the *LPL* locus are significantly associated with TG(52:2), TG(52:3), TG(53:2), and TG(53:3), which have a high probability of containing one or more MUFAs within their fatty acid side chains. Figure 2 also shows that triglycerides and diglycerides are predominantly inversely associated with *LPL* variants, while triglycerides are positively associated with patatin-like phospholipase domain containing protein 3 *(PNPLA3)* variants. Variants in the *LPL* locus are also positively associated with phosphocholines, sphingomyelins, and cholesterol esters, although the associations for the majority of the lipids in these subclasses did not reach genome-wide significance.

### New biological insights into lipid metabolism

Our analysis replicated known associations between lipids and genetic loci while also further extending what is known about these loci. We found significant associations of a wide range of lipids, including [PA(39:1)+H]^+^, [PC(35:4)+H]^+^, and [PE(36:4)+H]^+^, with variants in the *LIPC* locus (Supplementary Figure 7k); and significant associations of five specific sphingomyelins ([SM(34:0)+H]^+^, [SM(40:0)+H]^+^, [SM(40:1)+H]^+^, [SM(40:2)+H]^+^, and [SM(42:1)+H]^+^), and with variants in the *APOE-C1-C2-C4* locus (Supplementary Figure 7c). We also identified significant associations of four further sphingomyelins ([SM(32:1)-CH_3_]^-^, [SM(32:1)+H]^+^, [SM(32:1)+OAc]^-^, and [SM(39:1)+H]^+^) with variants in the *SGPP1* locus (Supplementary Figure 7u). Additionally, we found significant associations of nine ceramides ([Cer(40:0)-H]^-^, [Cer(40:1)-H]^-^, [Cer(40:2)-H]^-^, [Cer(41:0)-H]^-^, [Cer(41:1)-H]^-^, [Cer(41:2)-H]^-^, [Cer(42:0)-H]^-^, [Cer(42:1)-H]^-^, and [Cer(42:2)-H]^-^) with variants in the *SPTLC3* locus, which have not previously been reported in relation to this locus, as well as significant associations with three phosphatidylcholines and fifteen sphingomyelins (Supplementary Figure 7v).

We also discovered genetic associations with lipids at *PNPLA3* and membrane bound *O*-acyltransferase domain containing 7 (*MBOAT7*) loci that may have important biological and clinical implications. We found significant associations of two triglycerides—[TG(56:6)+NH_4_]^+^ (*m/z* 924.801) and [TG(56:5)+NH_4_]^+^ (*m/z* 926.817)—with rs12484809, an intronic variant in the *PNPLA3* locus (Supplementary Figure 7s). We also found that the lead SNP in the *MBOAT7* locus, rs8736 (chr19:54677189), was associated with a wide range of phosphatic acids (e.g. [PA(40:5)+OAc]^-^ and [PA(44:6)+OAc]^-^), phosphatidylcholines (e.g. [PC(36:6)+OAc]^-^ and [PC(42:11)+OAc]^-^), and phosphoinositols (e.g. [PI(34:1)-H]^-^) (Supplementary Figure 7m).

We undertook further investigation of a related nonsynonymous *PNPLA3* variant that is in moderate LD (*r*^2^ = 0.695), rs738409 (p.Ile148Met), to study the associations of lipids with *PNPLA3* in greater detail, including those that did not reach genome-wide significance. We focused on this variant rather than rs12484809 because I148M is already known to be associated with total triglycerides [33] and has been extensively characterised in previous genetic and functional analyses, and therefore is more likely to have potential clinical applications. As shown in Fig. 5a, the *PNPLA3* I148M allele was associated with increased levels of lipids of higher carbon number and double-bond content, and consistently, with decreased levels of lipids of lower carbon number and double-bond content. There were also significant differences between the mean levels of the triglycerides TG(57:10), TG(46:0), and TG(56:6) between individuals stratified by *PNPLA3* I148M genotype (Fig. 5b–d).

## Discussion

Based on a comprehensive analysis of genetic influences on 340 human blood lipids assayed in 5662 individuals from Pakistan, we identified 253 significant associations between 181 lipids and 24 genetic loci. Additionally, in our analysis of 399 lipids in 13,814 British blood donors, we identified significant associations between 244 lipids and 38 independent loci. The majority of genetic regions associated with lipids in PROMIS were also found in INTERVAL; those that did not replicate may be due to the increased sample size in INTERVAL which gave a substantial boost in power. These findings suggest that genetically determined aspects of lipid metabolism are broadly similar in individuals of South Asian and European ancestry, and that DIHRMS can reliably capture differences in lipid levels across diverse populations.

There were six genetic loci specific to lipid levels in PROMIS: *ANGPTL3*, *UGT8*, *PCTP*, *C19orf80*, *XBP1*, and *GAL3ST1*. Angiopoietin-like 3 (*ANGPTL3*) is involved in the regulation of lipid and glucose metabolism. SNPs in the *ANGPTL3* region have previously been shown to be associated with major lipids, including LDL-C and total cholesterol [34, 35]. In PROMIS, rs6657050, an intronic variant in the *ANGPTL3* locus, was significantly associated with [PI(36:2)-H]^-^ (*m/z* 861.5498) (Supplementary Figure 7a).

UDP glycosyltransferase 8 (*UGT8*) catalyses the transfer of galactose to ceramide, a key enzymatic step in the biosynthesis of galactocerebrosides, which are abundant sphingolipids of the myelin membrane of the central and peripheral nervous system. In PROMIS, rs28870381, an intergenic variant in *UGT8*, was associated with [PG(32:1)+OAc]^-^ (*m/z* 779.5078) (Supplementary Figure 7w).

Phosphatidylcholine transfer protein (*PCTP*) catalyses the transfer of phosphatidylcholines between membranes and is involved in lipid binding. Through regulation of plasma lipid concentrations, it may also modulate the development of atherosclerosis [36]. In PROMIS, rs11079173, an intronic variant in the *PCTP* locus, was associated with [PA(40:5)+OAc]^-^ (*m/z* 809.5337) (Supplementary Figure 7p).

*C19orf80*, also known as angiopoietin-like 8 (*ANGPTL8*), is involved in the regulation of serum triglyceride levels and is associated with major lipids including HDL-C and triglycerides [35]. In PROMIS, rs8101801, an intronic variant in the *C19orf80* locus, was significantly associated with [PI(38:4)-H]^-^ (*m/z* 885.5498) (Supplementary Figure 7d).

Galactose-3-*O*-sulfotransferase 1 (*GAL3ST1*) catalyses the sulfation of membrane glycolipids and the synthesis of galactosylceramide sulfate, a major lipid component of the myelin sheath. In PROMIS, rs2267161, a missense variant in the *GAL3ST1* locus, was associated with [PG(32:1)+OAc]^-^ (*m/z* 779.5078) (Supplementary Figure 7i).

X-box binding protein 1 (*XBP1*) functions as a transcription factor during endoplasmic reticulum stress by regulating the unfolded protein response. It is also a major regulator of the unfolded protein response in obesity-induced insulin resistance and T2D for the management of obesity and diabetes prevention. Recent studies have shown that compounds targeting the *XBP1* pathway are a potential approach for the treatment of metabolic diseases [37]. In addition, *XBP1* protein expression, which is induced in the liver by a high carbohydrate diet, is directly involved in fatty acid synthesis through de novo lipogenesis. Therefore, compounds that inhibit *XBP1* activation may also be useful for the treatment of NAFLD [38]. In PROMIS, rs71661463, an intronic variant for which *XBP1* is the candidate causal gene, was associated with [SM(37:1)+H]^+^ (*m/z* 745.6216) (Supplementary Figure 7x). Recent research across many species has shown that *XBP1* is a transcription factor regulating hepatic lipogenesis. In mice, hepatic *XBP1* expression is regulated by proopiomelanocortin (POMC) during sensory food perception and coincides with changes in the lipid composition of the liver with increases in PCs and PEs [39]. Although previous studies have shown direct links between *XBP1* and overall lipid metabolism, this is the first time a genetic association has been reported between *XBP1* and lipid metabolites in humans, affecting sphingomyelins, PCs, and PEs (Supplementary Figure 7x).

Our findings for the *PNPLA3* and *MBOAT7* loci were also notable. *PNPLA3* is a multifunctional enzyme that encodes a triacylglycerol lipase, which mediates triacylglycerol hydrolysis in adipocytes and has acylglycerol *O*-acyltransferase activity. The relationship between rs738409, a nonsynonymous variant (p.Ile148Met) in the *PNPLA3* gene, and non-alcoholic fatty liver disease (NAFLD) has been well established [40]. This variant has been shown to impair triglyceride hydrolysis in the liver and secretion of triglyceride-rich very low-density lipoproteins, leading to the altered fatty acid composition of liver triglycerides, and is also associated with reduced risk of CHD [41] and increased risk of type 2 diabetes (T2D) [42]. This suggests that targeting hepatic pathways to reduce cardiovascular risk may be complex, despite the clustering of cardiovascular and hepatic diseases in people with metabolic syndrome. Our analysis offers granularity to the previously identified total triglyceride associations with *PNPLA3* by identifying two specific triglyceride species that may have a role in *PNPLA3* function.

*MBOAT7*, which contributes to the regulation of free arachidonic acid in the cell through the remodelling of phospholipids, was reported as being associated with the metabolite 1-arachidonoylglycerophosphoinositol in a previous mGWAS [19] (known as [PI(36:4)-H]^-^ in our study), but we found that the lead SNP in this locus, rs8736 (chr19:54677189), was also associated with a wide range of phosphatic acids, phosphatidylcholines, phosphatidylethanolamines, and phosphoinositols (Supplementary Figure 7m). Several studies have shown that *MBOAT7* (also known as lysophosphatidylinositol-acyltransferase 1 [*LPIAT1*]) is responsible for the transfer of arachidonoyl-CoA to lysophosphoinositides [43]. The creation of *MBOAT7*-deficient macrophages show a decreased level of [PI(38:4)-H]^-^ and an increase of [PI(34:1)-H]^-^ as well as [PI(40:5)-H]^-^ [44]. The T allele of rs8736, a 3’ UTR SNP, shows a similar shift in the phosphatidylinositol metabolism. Our work shows that this SNP is also strongly associated with [PI(38:3)+OAc]^-^ (*m/z* 947.5866), which is likely to be the dihomo-gamma linoleic acid (20:3n6)-containing phosphoinositol. None of the papers testing the substrate specificity of *MBOAT7* have included dihomo-gamma linoleic acid or [PI(38:3)+OAc]^-^ in their analysis. Thus, we provide novel evidence in humans that there is an association between *MBOAT7* activity and circulating phosphatidylinositols, a finding that requires further replication.

Our network diagram helped identify sphingomyelins that were associated exclusively with four loci that were not associated with any other lipid subclasses: *GCKR*, *SGPP1*, *MLXIPL*, and *XBP1*. Sphingomyelins have previously been shown to be associated with *SGPP1* [45], but the associations of sphingomyelins with these other three loci are reported here for the first time. *GCKR* has been shown to be associated with total cholesterol and triglycerides (see Fig. 2) and has also been associated with the plasma phospholipid fraction fatty acids 16:0 and 16:1 [46, 47]; most lipids that we found to be associated with *GCKR* (Supplementary Figure 7j) are likely to contain these particular fatty acids. It has been suggested that the glucokinase receptor, encoded by *GCKR*, affects the production of malonyl-CoA, an important substrate for de novo lipogenesis [46]. To a similar extent, there is a known relation between *MLXIPL* and carbohydrate and lipid metabolism. *MLXIPL* is a transcription factor affecting carbohydrate response element-binding protein (CREBP) and therefore also plays a role in lipogenesis. Although both these genes have previously been linked to lipogenesis, we discovered that genetic variation at genes involved in the regulation of lipogenesis has been implicated in altering sphingomyelin concentrations.

The network diagram also helped recapitulate known biological relationships between lipids. As we established in our previous analysis [7], the number of significant partial correlations between lipids of different subclasses was significantly higher than would be expected due to chance alone. This analysis further showed that genes that were significantly associated with lipids of a particular subclass regulated all of the lipids within the subclass in a similar manner. Therefore, the total concentrations of a given lipid class associated with a genetic locus are less affected by the proportion of fatty acids present in those lipid species.

In summary, our analyses resulted in the following new insights in an understudied South Asian population: (1) we established that decreased levels of sphingomyelins are associated with genetically lower *LPL* activity; (2) we revealed a wide range of glycerophospholipids that are associated with variants in the *MBOAT7* locus; (3) we identified several new associations of phosphatic acids, phosphocholines, and phosphoethanolamines with variants in the *LIPC* region; (4) we found several novel associations of sphingomyelins and phosphocholines with variants in the *APOE-C1-C2-C4* cluster; (5) we discovered four new associations of sphingomyelins with variants in the *SGPP1* locus; and (6) we found several previously unreported associations of phosphocholines, sphingomyelins, and ceramides with variants in the *SPTLC3* locus. These findings can help further the identification of novel therapeutic targets for prevention and treatment.

Our investigation into the genetic influences of lipids has several strengths. First, the research involved participants from a population cohort in Pakistan, thereby enhancing the scientific understanding of lipid associations in this understudied population, and we compared the findings with a typical Western population of British blood donors using the same lipid-profiling platform. Second, the analysis was based on a relatively large dataset of 5662 participants from Pakistan and an even larger cohort of 13,814 individuals from the UK, thereby increasing statistical power to detect associations. Third, our mGWAS was performed in individuals free from established MI at baseline in PROMIS and healthy blood donors in INTERVAL, which reduces spurious associations due to the disease state or potential treatments. Finally, our newly developed open-profiling lipidomics platform was utilised to provide detailed lipid profiles, with a wider coverage of lipids than most other high-throughput profiling methods [7], which improved our ability to detect novel associations and our understanding of the detailed effects of known lipid loci at the level of individual lipid species.

Nevertheless, our study has several technical limitations. To enable the rapid and robust lipid profiling of such a large number of samples, we employed DIHRMS. Despite the advantages of this platform, it is unable to distinguish isobaric lipids. This means that different lipid species can contribute to the same signal; for instance, [PC(32:1)+H]^+^ and [PE(35:1)+H]^+^ both have the same molecular formula (C_40_H_77_NO_8_P) and will both contribute to the signal of *m/z* 732.5541. Furthermore, even [PC(32:1)+H]^+^ consists of both PC(16:0/16:1) and PC(14:0/18:1). These limitations are discussed in detail in our previous methodological paper on this platform [7], while the relevance of using these aggregate of signals in metabolic studies has been shown by other studies [45]. Further work, with improved analytical resolution, will enable further pinpointing of the relevant lipids to the identified loci.

The cohorts included in our analysis also have several potential limitations. First, possible selection biases arise from the case-control design of PROMIS, although this was minimised by the recruitment of controls from patients, visitors of patients attending out-patient clinics, and unrelated visitors of cardiac patients. Second, serum samples in PROMIS were stored in freezers at −80°C for between 2 and 8 years before aliquots were taken for the lipidomics measurements, which we accounted for by adjusting the analyses by the number of years that the samples had been stored. Although residual confounding and deterioration of lipid profiles may still exist, such deterioration is unlikely to have been related to genotype. Third, a majority (76%) of PROMIS participants had not fasted prior to blood draw, and a small proportion of participants (7%) had reportedly fasted for an unknown duration. Recent food consumption may have had significant effects on lipid levels and influenced the results. Our analyses adjusted for fasting status although we lacked statistical power to stratify by fasting status. Fourth, PROMIS participants were recruited from multiple centres in urban Pakistan [7], but it is unclear whether the findings from this study would be generalizable to individuals living in rural villages and other parts of Pakistan, or in other countries in South Asia. However, the confirmatory analysis in INTERVAL, in which we identified significant associations with lipids for the majority of the genetic loci found in PROMIS, helps strengthen the argument that these findings are generalizable. Additionally, many of the lipids were associated with known genetic regions such as *APOA5-C3* and *FADS1-2-3*, which have already been shown to be associated with multiple lipids in other Western populations, further strengthening the validity of the findings from this analysis. Finally, although two-sample Mendelian randomization approaches to make causal inferences about the association of lipids with CHD risk factors and disease outcomes hold great promise in the lipidomics arena [48], extensive pleiotropy made it too difficult to disentangle the findings and we chose not to pursue this avenue. Therefore, although especially stringent procedures were followed, highly conservative cut-offs were used to determine statistical significance, and rigorous pre-analysis and post-analysis quality control steps were performed, there is still a possibility that some of the findings were false positives that arose due to artefacts rather than being true signals. Additional analyses in other populations using the DIHRMS lipidomics platform would be helpful to further replicate our findings. Moreover, the identified pathways and proposed molecular mechanisms require validation through functional analyses in model organisms and humans.

Further research will be able to leverage these lipidomics results in combination with whole-genome and whole-exome sequencing performed in PROMIS and INTERVAL to help understand the consequences of loss-of-function mutations identified in these participants [49].

## Conclusions

In conclusion, this article presents the results from a comprehensive analysis of genetic influences on human blood lipids in South Asians with a comparative analysis in the UK. Our findings strengthen and expand the knowledge base for understanding the genetic determinants of lipids and their association with cardiometabolic disease-related loci. These findings have important implications for the identification of novel therapeutic targets and advancement of mechanistic understanding of metabolic pathways that may lead to the onset of chronic diseases and lipid-related abnormalities.

## Supplementary Information

**Additional file 1.** Supplementary Methods; Supplementary Figures 1-8.

**Additional file 2.** Supplementary Tables 1-18.

**Additional file 3.** Supplementary Figure 1 (high resolution).

## Data Availability

The datasets used and/or analysed during the current study are available from the corresponding author on reasonable request.

## Abbreviations

CHD: Coronary heart disease
CVD: Cardiovascular disease
DHA: Docosahexaenoic acid
DIHRMS: Direct infusion high-resolution mass spectrometry
FDR: False discovery rate
GGM: Gaussian Graphical Model
HWE: Hardy-Weinberg Equilibrium
MAF: Minor allele frequency
MI: Myocardial infarction
*m/z*: Mass-charge ratio
NAFLD: Non-alcoholic fatty liver disease
PROMIS: Pakistan Risk of Myocardial Infarction Study
PUFA: Polyunsaturated fatty acid
SD: Standard deviation
SNP: Single nucleotide polymorphism
T2D: Type 2 diabetes
QC: Quality control
QTL: Quantitative trait loci
