# Supplementary material for: Genome-wide analysis of blood lipid metabolites in over 5000 South Asians reveals biological insights at cardiometabolic disease loci
Source: BMC Med. 2021 Sep 10;19:232. doi: 10.1186/s12916-021-02087-1 (PMC8431908; doi:10.1186/s12916-021-02087-1)
Supplement: Supplementary file 1 — Additional file 1. Supplementary Methods; Supplementary Figures 1-8. [file 12916_2021_2087_MOESM1_ESM.pdf]

Genome-wide analysis of blood lipid metabolites in over 5000 South Asians reveals biological insights at cardiometabolic disease loci

SUPPLEMENTARY MATERIAL

Supplementary Tables

Supplementary Table 1. Lipids measured in PROMIS

Supplementary Table 2. Lipids measured in INTERVAL

Supplementary Table 3. Number of significant variants and loci associated with each lipid subclass

Supplementary Table 4. Summary of significant associations between lipid metabolites and genetic variants from univariate genome-wide association study in PROMIS

Supplementary Table 5. Summary of significant associations between lipid metabolites and genetic variants from conditional analyses of univariate genome-wide association study results in PROMIS

Supplementary Table 6. Summary of significant associations between lipid metabolites and genetic variants from univariate genome-wide association study in INTERVAL

Supplementary Table 7. Summary and classification of analysed ratios in PROMIS

Supplementary Table 8. Summary of significant associations between ratios of lipid metabolites and genetic variants from univariate genome-wide association study in PROMIS

Supplementary Table 9. Prediction of causal genes in PROMIS based on integration of information from bottom-up and top-down SNP annotation approaches

Supplementary Table 10. Annotation of genome-wide significant associations in PROMIS from the level of the variant (bottom-up)

Supplementary Table 11. Annotation of genome-wide significant associations in PROMIS by proximal (±500-Kb from lead variant) gene function (top-down)

Supplementary Table 12. Lead variants in PROMIS residing in exonic sequence

Supplementary Table 13. Lead variants in PROMIS in high linkage disequilibrium ($r^2 \geq 0.8$) with ≥ 1 non-synonymous SNP

Supplementary Table 14. Lead SNP *cis*-eQTLs in PROMIS in lipid-relevant human tissues

Supplementary Table 15. Enrichment analysis of cell-type specific enhancer overlap with our set of 90 lead SNPs from conditional analyses in PROMIS using HaploReg v4.1

Supplementary Table 16. Prediction of causal genes in INTERVAL based on integration of information from bottom-up and top-down SNP annotation approaches

Supplementary Table 17. Summary of associations between lipid metabolites in PROMIS and 175 major lipid loci

Supplementary Table 18. Summary of significant associations between lipid metabolites and genetic variants from univariate genome-wide association study in PROMIS with adjustment for clinical lipid measures

Supplementary Figures

Supplementary Figure 1. Extended heat map showing associations of significant loci from conditional analyses in PROMIS with all lipid metabolites, major lipids, and lipid-related diseases/disorders

Supplementary Figure 2. Genetic architecture of serum lipid levels in PROMIS

Supplementary Figure 3. Comparison of associations in PROMIS and INTERVAL

Supplementary Figure 4. Number of lipids in PROMIS associated with each variant

Supplementary Figure 5. Increased *de novo* lipogenesis in lipodystrophy and NAFLD patients

Supplementary Figure 6. Flow diagram outlining strategy for mediating gene prioritisation

Supplementary Figure 7. Association of lipids in PROMIS with significantly associated loci from conditional analyses

Supplementary Figure 8. Comparison of genetic associations with lipids pre- and post-adjustment for clinical lipid measures

SUPPLEMENTARY FIGURES

Supplementary Figure 1. Extended heat map showing associations of significant loci from conditional analyses in PROMIS with all lipid metabolites, major lipids, and lipid-related diseases/disorders

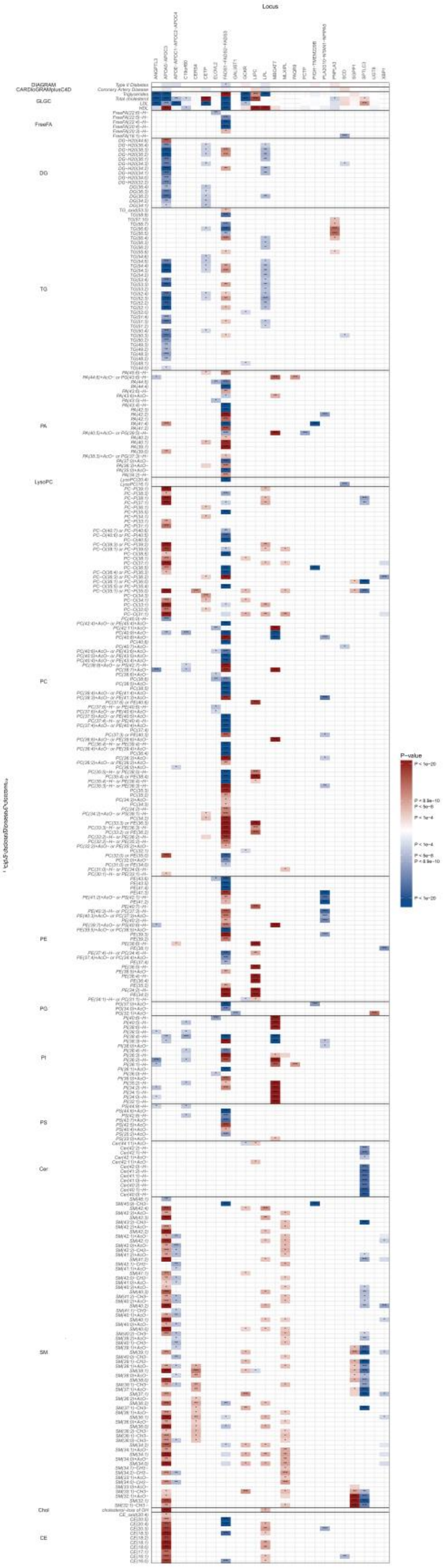

Caption for Supplementary Figure 1 (previous page):
The effect estimates of the associations between significant variants with all lipid metabolites in PROMIS are plotted as a heat map. Results are shown for the association of all lipid metabolites (rows) with the most strongly associated genetic variant within each locus (columns). The associations with major lipids from the GLGC (total cholesterol, HDL-C, LDL-C, and triglycerides), DIAGRAM Consortium (type 2 diabetes), and CARDIoGRAMplusC4D Consortium (coronary artery disease) are also shown. The magnitude and direction of the effect estimates (standardised per 1-SD) are indicated by a colour scale, with blue indicating a negative association and red indicating a positive association with respect to the SNP effect on the trait. Asterisks indicates the degree of significance of the *P*-values of association. * = $P < 1 \times 10^{-4}$; ** = $P < 5 \times 10^{-8}$; *** = $P < 8.9 \times 10^{-10}$. **Note:** A high-resolution version of this figure is available as Additional file 3.

Supplementary Figure 2. Genetic architecture of serum lipid levels in PROMIS

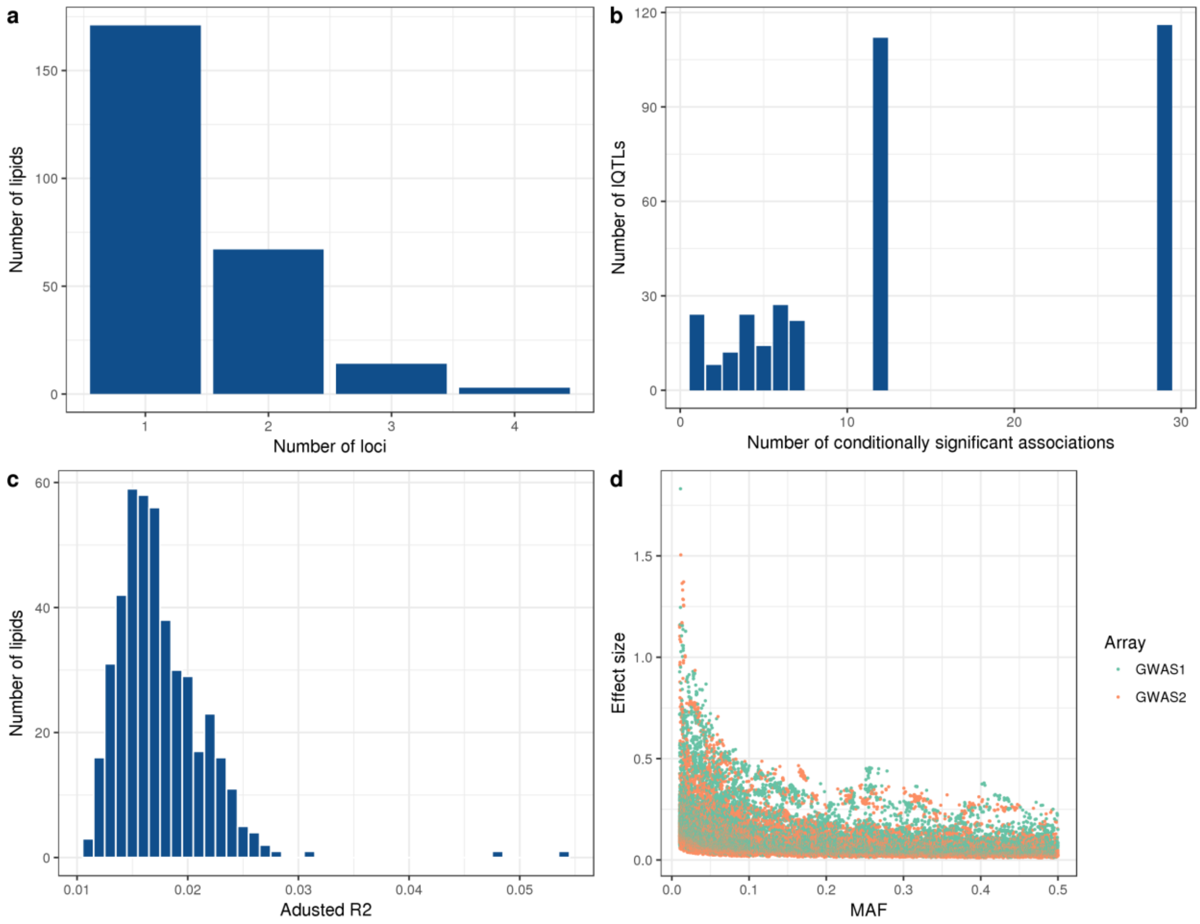

n = 5,662 participants from PROMIS. (a) Number of significantly associated loci per lipid. (b) Number of conditionally significant associations within each lipid QTL. (c) Histogram of variance explained by conditionally independent variants. (d) Effect size versus MAF.

Supplementary Figure 3. Comparison of associations in PROMIS and INTERVAL

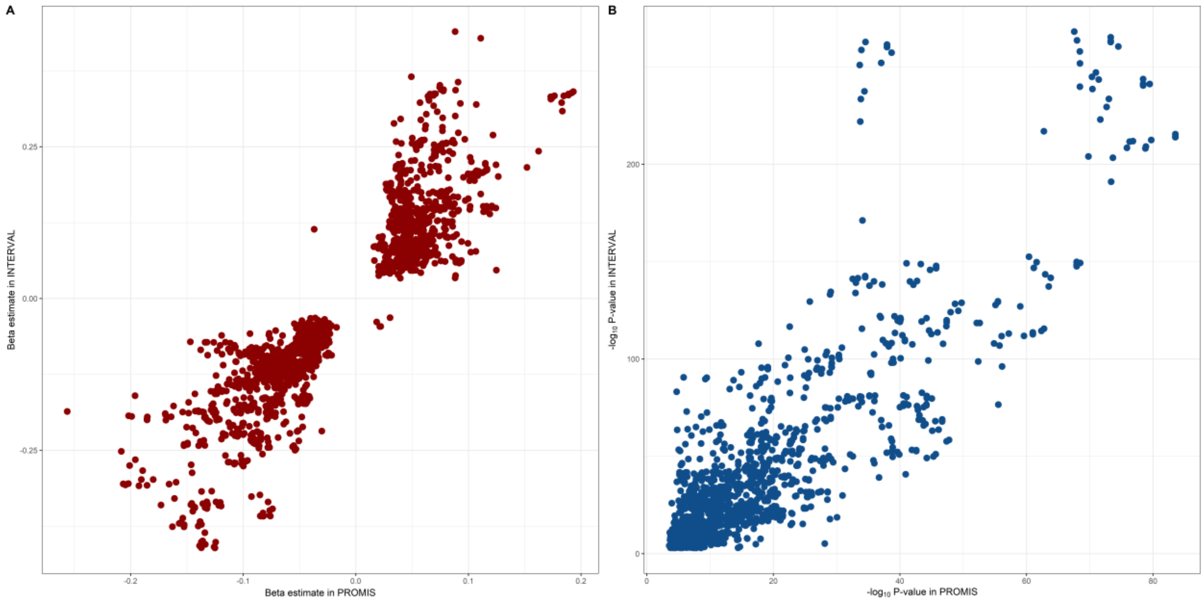

(a) Beta estimates in INTERVAL vs PROMIS. (b) $-\log_{10}$ *P*-values in INTERVAL vs PROMIS.

Supplementary Figure 4. Number of lipids in PROMIS associated with each variant

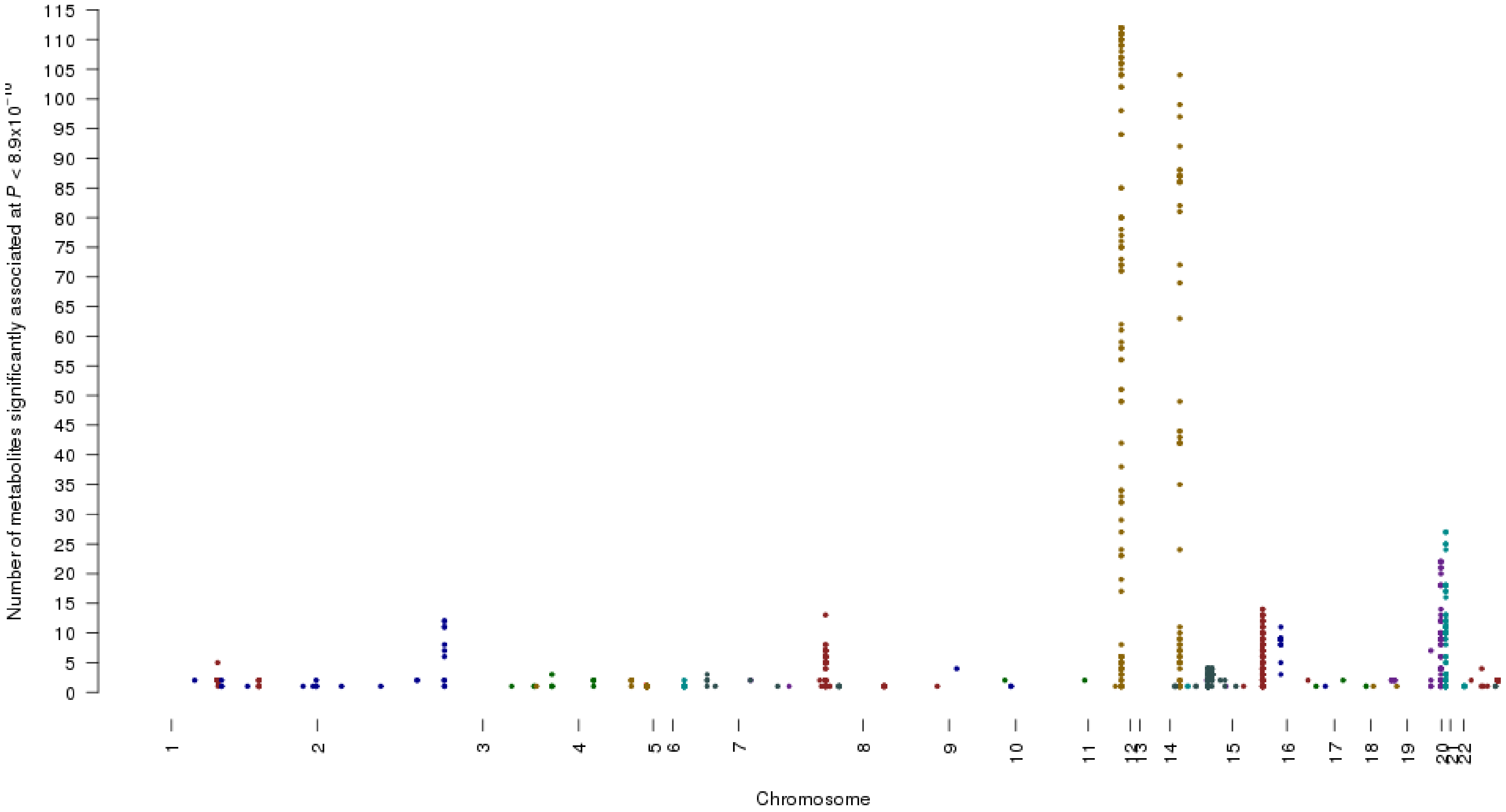

Results are shown for the number of lipids in PROMIS associated with each variant at genome-wide significance ($P < 8.9 \times 10^{-10}$).

Supplementary Figure 5. Increased *de novo* lipogenesis in lipodystrophy and NAFLD patients

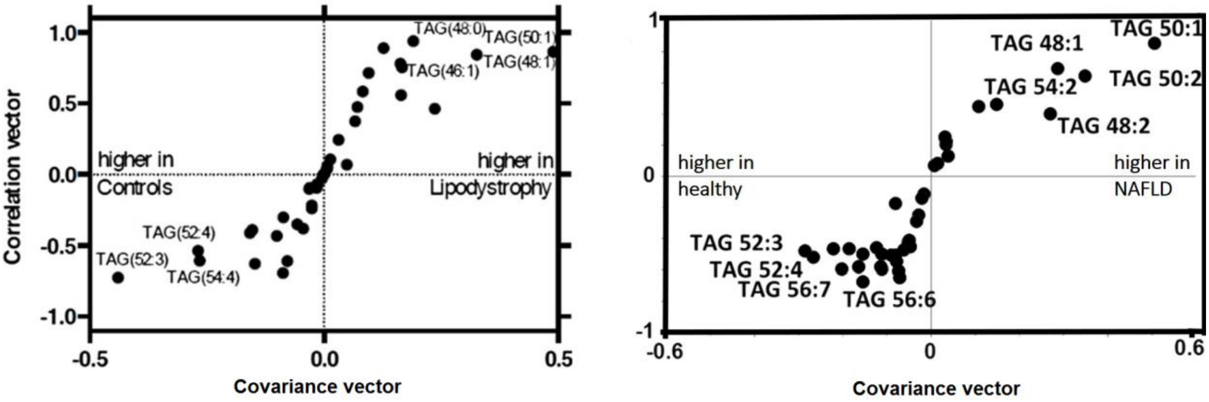

Increased *de novo* lipogenesis in lipodystrophy patients (left panel based on Eiden et al 2015) [49] and NAFLD patients (right panel based on Sanders et al 2018) [50] both show an increase in TAG(48:1) and TG(50:1) originating from the liver, leading to lower levels of TG(52:4) and other triglycerides associated with *APOA5-C3* variants.

Supplementary Figure 6. Flow diagram outlining strategy for mediating gene prioritisation

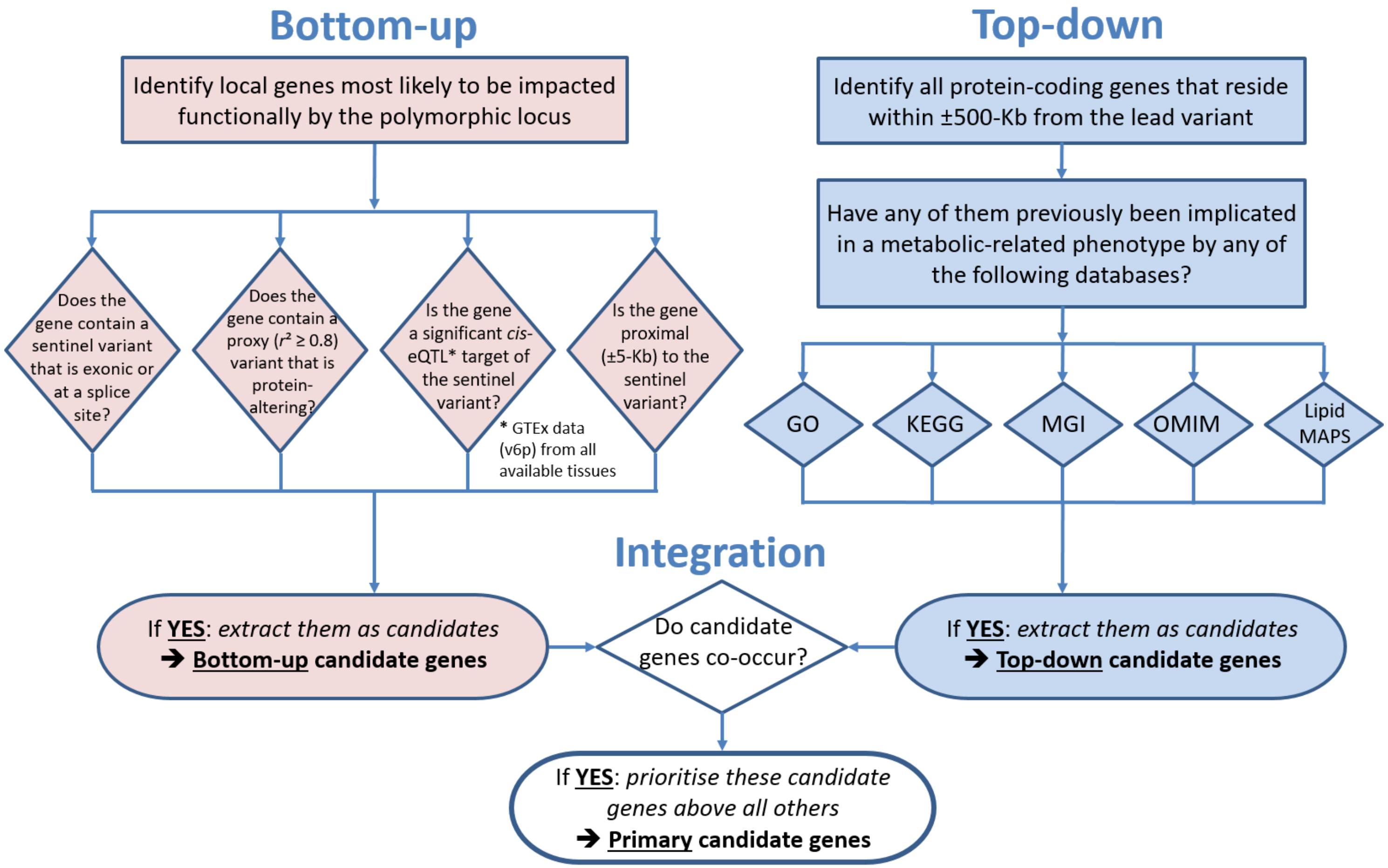

The flow diagram shows how the "bottom-up" and "top-down" approaches were used and then integrated to identify probable causal genes for each significantly associated variant. A proxy is defined as those variants with $r^2 \geq 0.8$ with the lead (EUR population, 1000 Genomes). **Abbreviations: eQTL** = Expression Quantitative Trait Locus; **GO** = Gene Ontology; **GTEx** = Genotype-Tissue Expression; **KEGG** = Kyoto Encyclopedia of Genes and Genomes; **Lipid MAPS** = Lipid Metabolites and Pathways Strategy; **MGI** = Mouse Genome Informatics; **OMIM** = Online Inheritance in Man. Adapted from Stacey et al [14].

Supplementary Figure 7. Association of lipids in PROMIS with significantly associated loci from conditional analyses

(a) *ANGPTL3*

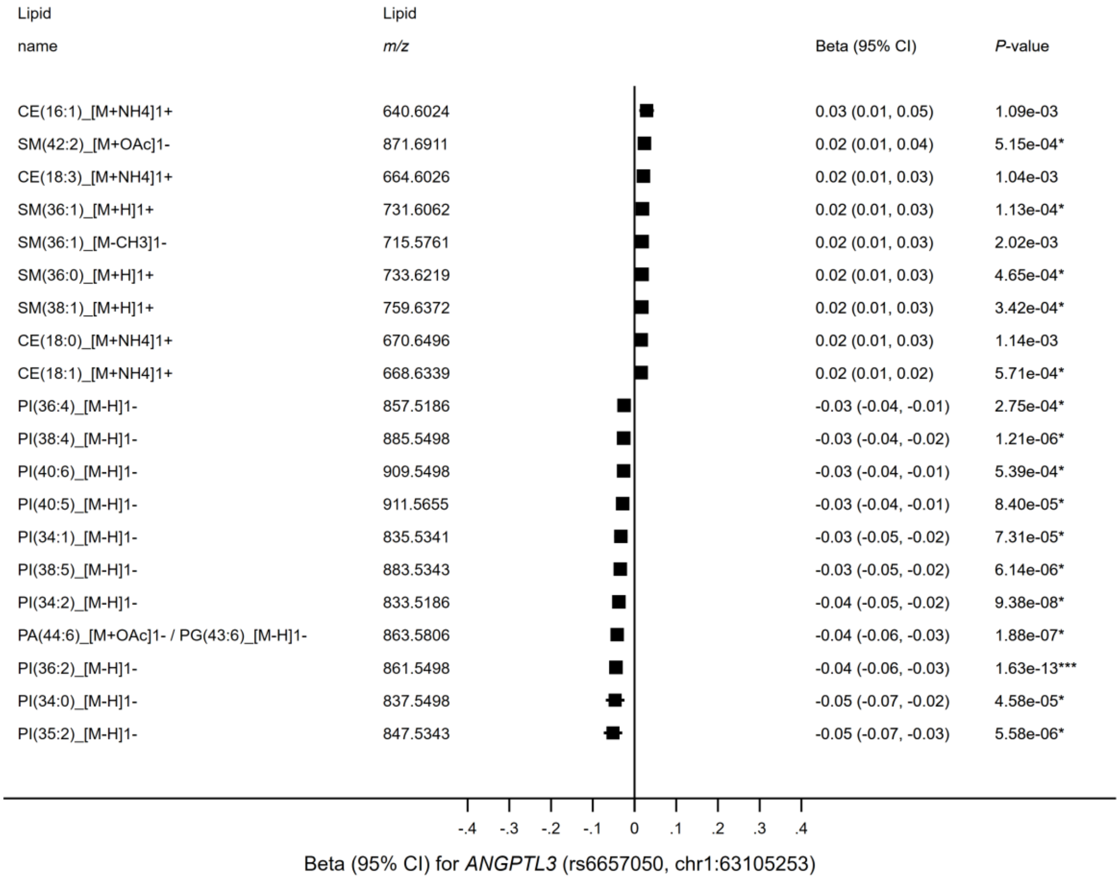

(b) *APOA5-APOC3*

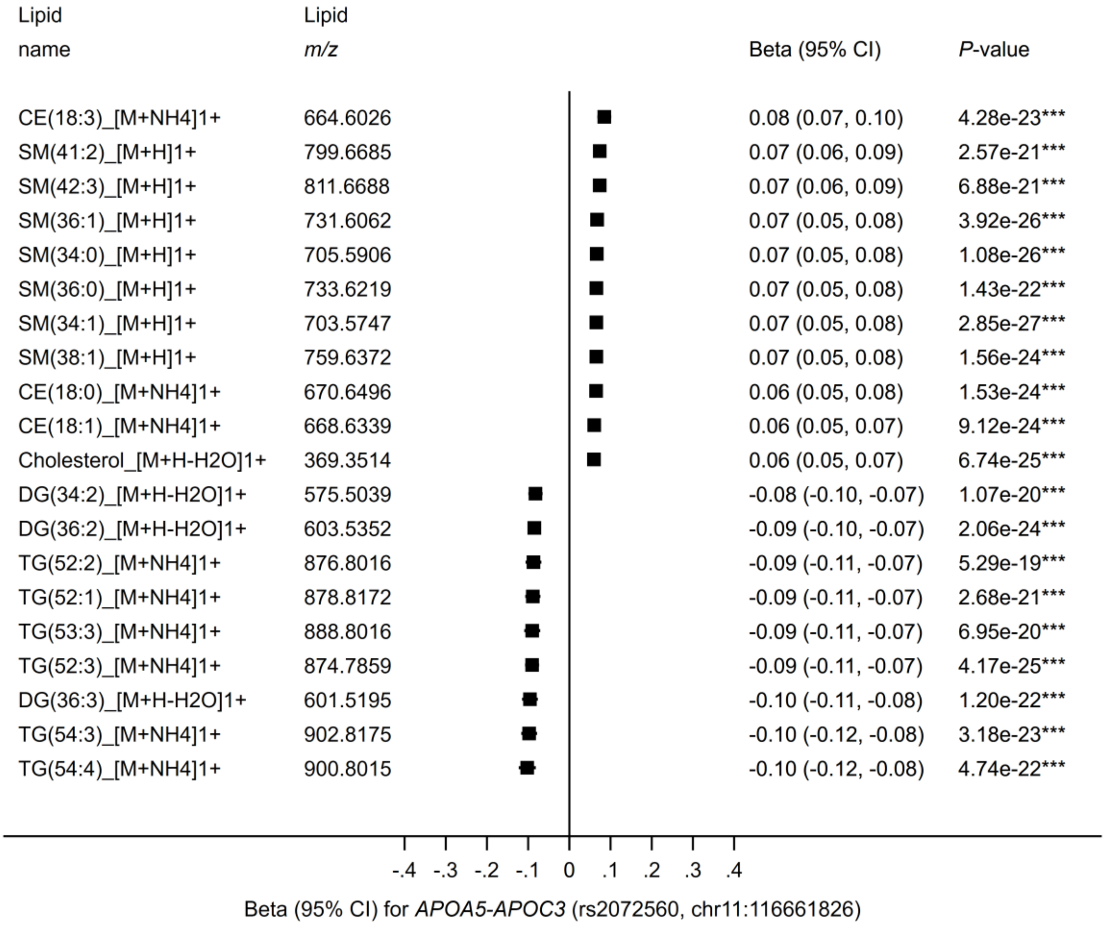

(c) ***APOE-APOC1-APOC2-APOC4***

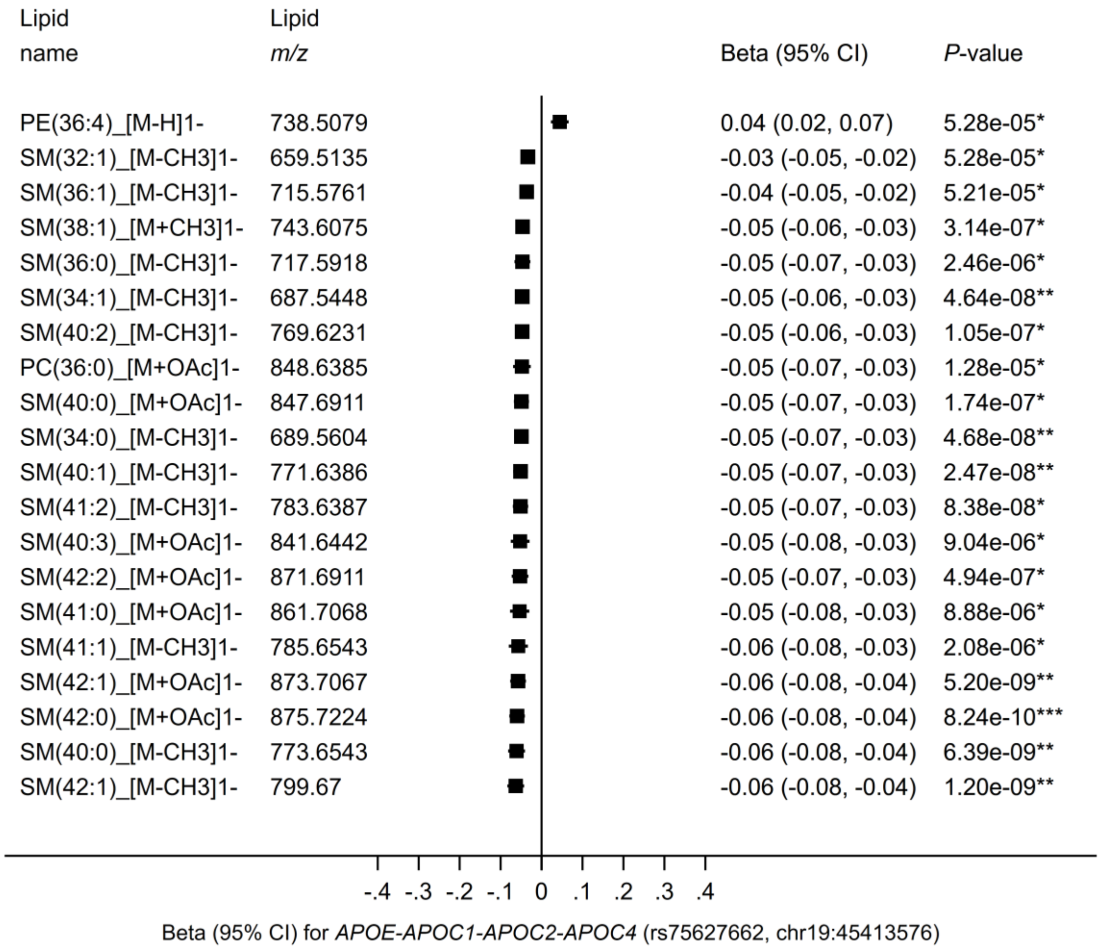

(d) *C19orf80*

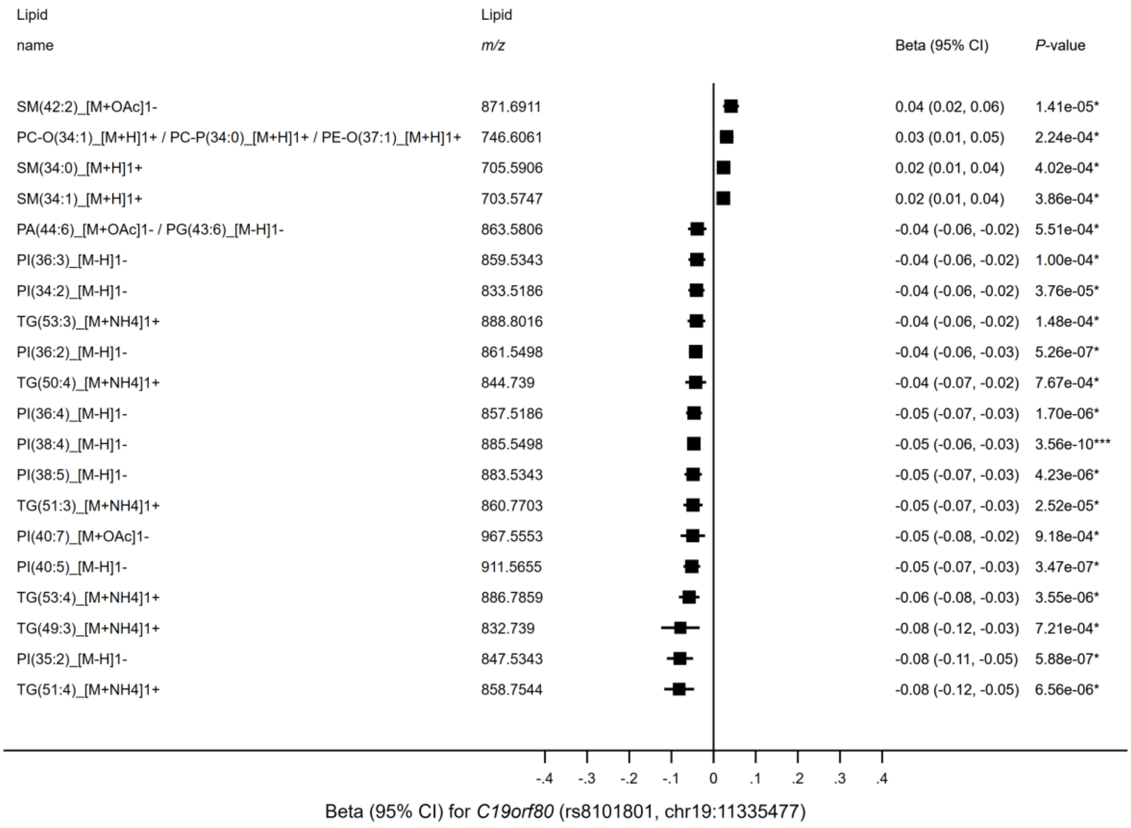
Lipid name
Lipid m/z
Beta (95% CI)
P-value
SM(42:2)_[M+OAc]1- 871.6911 0.04 (0.02, 0.06) 1.41e-05*
PC-O(34:1)_[M+H]1+ / PC-P(34:0)_[M+H]1+ / PE-O(37:1)_[M+H]1+ 746.6061 0.03 (0.01, 0.05) 2.24e-04*
SM(34:0)_[M+H]1+ 705.5906 0.02 (0.01, 0.04) 4.02e-04*
SM(34:1)_[M+H]1+ 703.5747 0.02 (0.01, 0.04) 3.86e-04*
PA(44:6)_[M+OAc]1- / PG(43:6)_[M-H]1- 863.5806 -0.04 (-0.06, -0.02) 5.51e-04*
PI(36:3)_[M-H]1- 859.5343 -0.04 (-0.06, -0.02) 1.00e-04*
PI(34:2)_[M-H]1- 833.5186 -0.04 (-0.06, -0.02) 3.76e-05*
TG(53:3)_[M+NH4]1+ 888.8016 -0.04 (-0.06, -0.02) 1.48e-04*
PI(36:2)_[M-H]1- 861.5498 -0.04 (-0.06, -0.03) 5.26e-07*
TG(50:4)_[M+NH4]1+ 844.739 -0.04 (-0.07, -0.02) 7.67e-04*
PI(36:4)_[M-H]1- 857.5186 -0.05 (-0.07, -0.03) 1.70e-06*
PI(38:4)_[M-H]1- 885.5498 -0.05 (-0.06, -0.03) 3.56e-10***
PI(38:5)_[M-H]1- 883.5343 -0.05 (-0.07, -0.03) 4.23e-06*
TG(51:3)_[M+NH4]1+ 860.7703 -0.05 (-0.07, -0.03) 2.52e-05*
PI(40:7)_[M+OAc]1- 967.5553 -0.05 (-0.08, -0.02) 9.18e-04*
PI(40:5)_[M-H]1- 911.5655 -0.05 (-0.07, -0.03) 3.47e-07*
TG(53:4)_[M+NH4]1+ 886.7859 -0.06 (-0.08, -0.03) 3.55e-06*
TG(49:3)_[M+NH4]1+ 832.739 -0.08 (-0.12, -0.03) 7.21e-04*
PI(35:2)_[M-H]1- 847.5343 -0.08 (-0.11, -0.05) 5.88e-07*
TG(51:4)_[M+NH4]1+ 858.7544 -0.08 (-0.12, -0.05) 6.56e-06*
-.4 -.3 -.2 -.1 0 .1 .2 .3 .4
Beta (95% CI) for C19orf80 (rs8101801, chr19:11335477)

(e) *CERS4*

Lipid name	Lipid *m/z*	Beta (95% CI)	*P*-value
TG(59:12)_[M+NH4]1+	954.754	0.18 (0.04, 0.32)	1.29e-02
TG(57:9)_[M+NH4]1+	932.7697	0.08 (0.01, 0.15)	2.67e-02
SM(42:8)_[M+OAc]1-	859.5972	0.05 (0.02, 0.08)	1.52e-03
SM(38:1)_[M+H]1+	759.6372	0.04 (0.03, 0.05)	1.65e-12***
SM(38:1)_[M+CH3]1-	743.6075	0.04 (0.02, 0.05)	6.76e-09**
SM(38:0)_[M+H]1+	761.6532	0.04 (0.02, 0.05)	2.43e-07*
SM(37:1)_[M+H]1+	745.6216	0.03 (0.02, 0.05)	1.41e-07*
SM(37:1)_[M-CH3]1-	729.5917	0.03 (0.02, 0.05)	1.16e-05*
SM(36:2)_[M-CH3]1-	713.5605	0.03 (0.02, 0.05)	5.28e-07*
PS(41:5)_[M+OAc]1-	910.5816	0.03 (0.01, 0.06)	1.48e-02
SM(36:2)_[M+H]1+	729.5906	0.03 (0.02, 0.04)	2.41e-08**
SM(36:0)_[M-CH3]1-	717.5918	0.03 (0.02, 0.04)	6.89e-06*
SM(36:1)_[M-CH3]1-	715.5761	0.03 (0.02, 0.04)	8.75e-07*
SM(36:1)_[M+H]1+	731.6062	0.03 (0.02, 0.04)	4.88e-08**
PC-O(33:2)_[M+H]1+ / PC-P(33:1)_[M+H]1+	730.5747	0.03 (0.01, 0.04)	4.11e-04*
SM(36:0)_[M+H]1+	733.6219	0.02 (0.01, 0.03)	7.01e-06*
SM(36:3)_[M+OAc]1-	785.5816	0.02 (0.00, 0.04)	4.87e-02
SM(39:1)_[M-CH3]1-	757.623	0.02 (0.00, 0.03)	3.78e-02
PC(40:4)_[M+OAc]1-	900.6698	0.02 (0.00, 0.03)	3.70e-02
SM(39:1)_[M+H]1+	773.6531	0.01 (0.00, 0.03)	3.39e-02

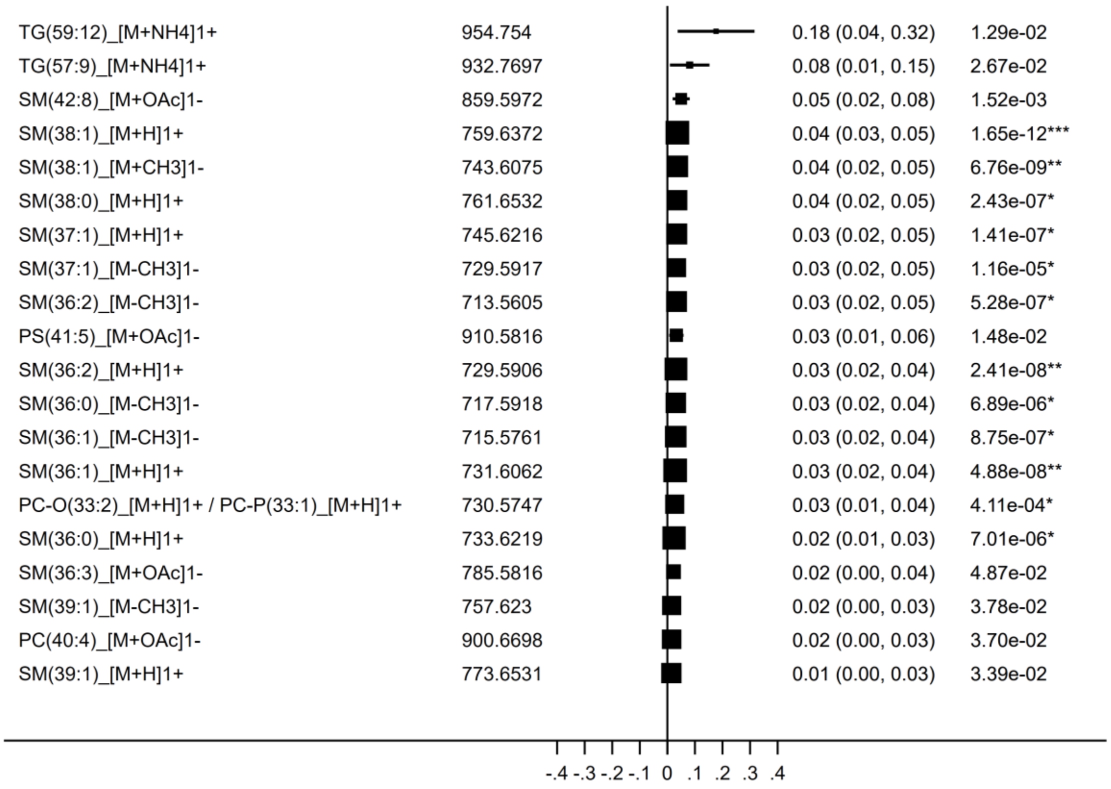

Beta (95% CI) for *CERS4* (rs11666866, chr19:8285607)

(f) *CETP*

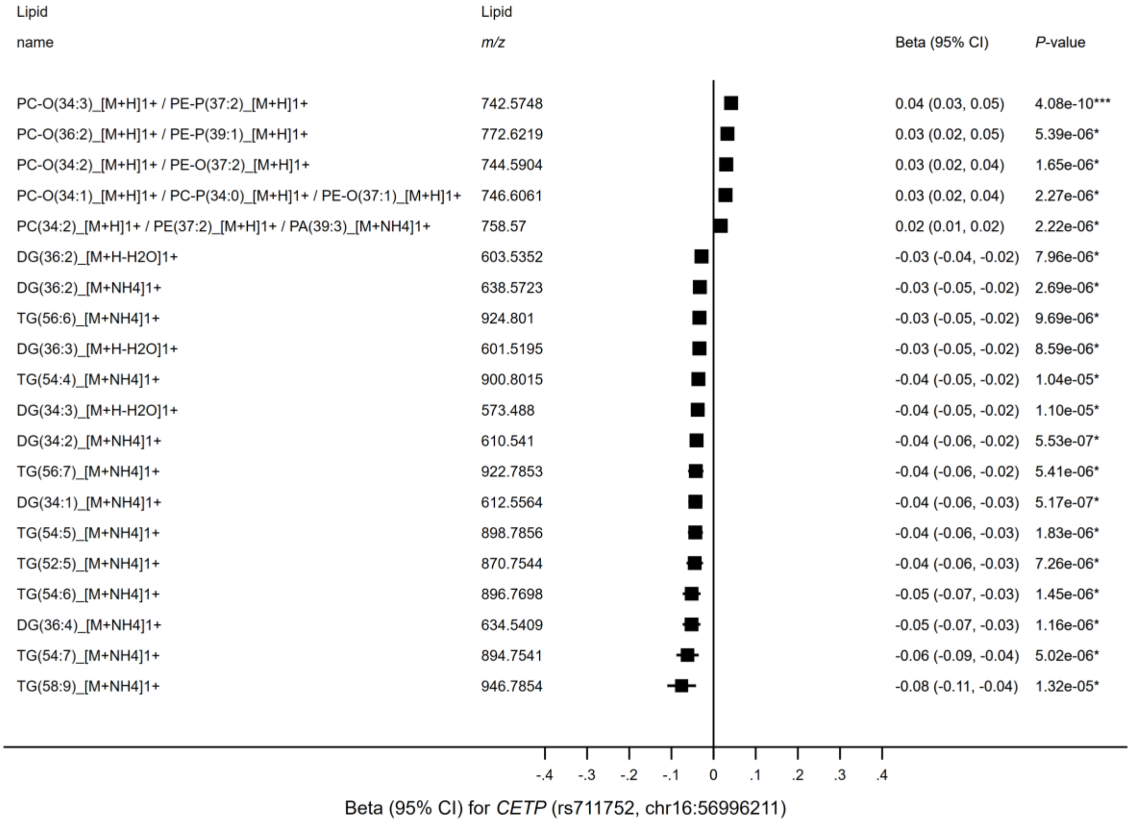
Lipid name
Lipid m/z
Beta (95% CI)
P-value
PC-O(34:3)_[M+H]1+ / PE-P(37:2)_[M+H]1+ 742.5748 0.04 (0.03, 0.05) 4.08e-10***
PC-O(36:2)_[M+H]1+ / PE-P(39:1)_[M+H]1+ 772.6219 0.03 (0.02, 0.05) 5.39e-06*
PC-O(34:2)_[M+H]1+ / PE-O(37:2)_[M+H]1+ 744.5904 0.03 (0.02, 0.04) 1.65e-06*
PC-O(34:1)_[M+H]1+ / PC-P(34:0)_[M+H]1+ / PE-O(37:1)_[M+H]1+ 746.6061 0.03 (0.02, 0.04) 2.27e-06*
PC(34:2)_[M+H]1+ / PE(37:2)_[M+H]1+ / PA(39:3)_[M+NH4]1+ 758.57 0.02 (0.01, 0.02) 2.22e-06*
DG(36:2)_[M+H-H2O]1+ 603.5352 -0.03 (-0.04, -0.02) 7.96e-06*
DG(36:2)_[M+NH4]1+ 638.5723 -0.03 (-0.05, -0.02) 2.69e-06*
TG(56:6)_[M+NH4]1+ 924.801 -0.03 (-0.05, -0.02) 9.69e-06*
DG(36:3)_[M+H-H2O]1+ 601.5195 -0.03 (-0.05, -0.02) 8.59e-06*
TG(54:4)_[M+NH4]1+ 900.8015 -0.04 (-0.05, -0.02) 1.04e-05*
DG(34:3)_[M+H-H2O]1+ 573.488 -0.04 (-0.05, -0.02) 1.10e-05*
DG(34:2)_[M+NH4]1+ 610.541 -0.04 (-0.06, -0.02) 5.53e-07*
TG(56:7)_[M+NH4]1+ 922.7853 -0.04 (-0.06, -0.02) 5.41e-06*
DG(34:1)_[M+NH4]1+ 612.5564 -0.04 (-0.06, -0.03) 5.17e-07*
TG(54:5)_[M+NH4]1+ 898.7856 -0.04 (-0.06, -0.03) 1.83e-06*
TG(52:5)_[M+NH4]1+ 870.7544 -0.04 (-0.06, -0.03) 7.26e-06*
TG(54:6)_[M+NH4]1+ 896.7698 -0.05 (-0.07, -0.03) 1.45e-06*
DG(36:4)_[M+NH4]1+ 634.5409 -0.05 (-0.07, -0.03) 1.16e-06*
TG(54:7)_[M+NH4]1+ 894.7541 -0.06 (-0.09, -0.04) 5.02e-06*
TG(58:9)_[M+NH4]1+ 946.7854 -0.08 (-0.11, -0.04) 1.32e-05*
-.4 -.3 -.2 -.1 0 .1 .2 .3 .4
Beta (95% CI) for CETP (rs711752, chr16:56996211)

(g) *ELOVL2*

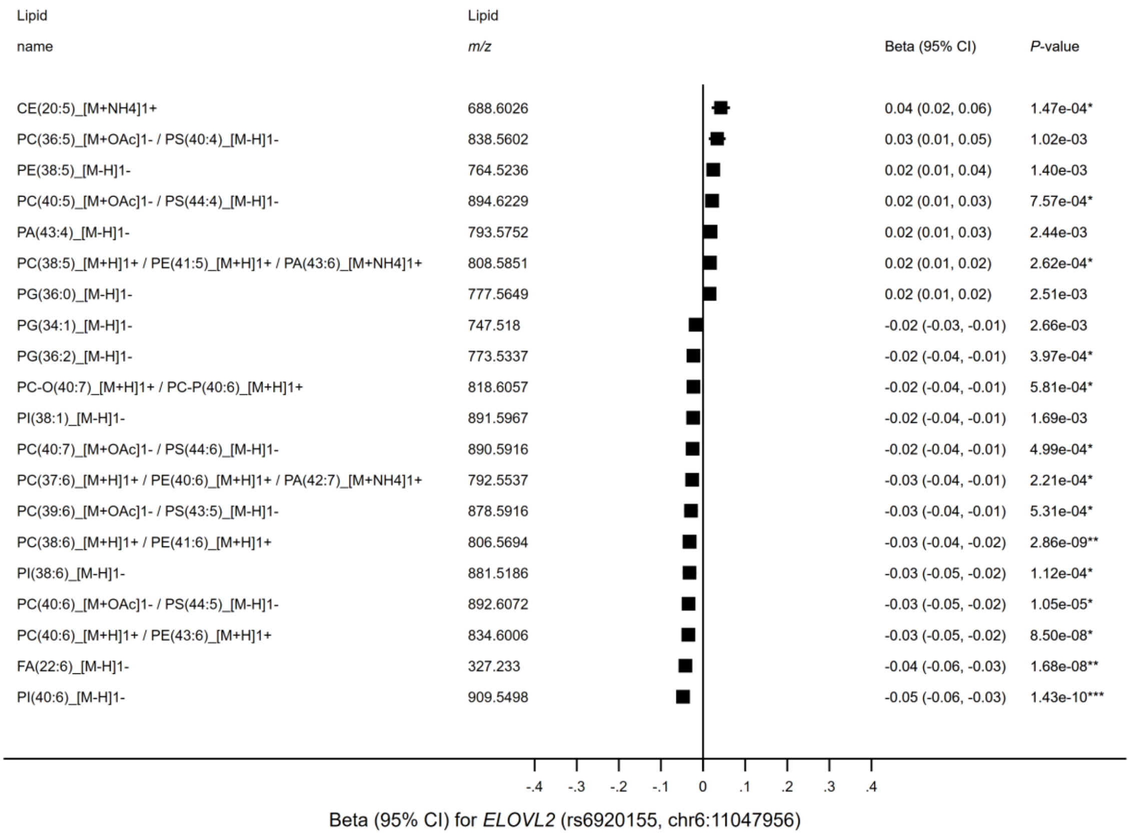
Lipid name
Lipid m/z
Beta (95% CI)
P-value
CE(20:5)_[M+NH4]1+ 688.6026 0.04 (0.02, 0.06) 1.47e-04*
PC(36:5)_[M+OAc]1- / PS(40:4)_[M-H]1- 838.5602 0.03 (0.01, 0.05) 1.02e-03
PE(38:5)_[M-H]1- 764.5236 0.02 (0.01, 0.04) 1.40e-03
PC(40:5)_[M+OAc]1- / PS(44:4)_[M-H]1- 894.6229 0.02 (0.01, 0.03) 7.57e-04*
PA(43:4)_[M-H]1- 793.5752 0.02 (0.01, 0.03) 2.44e-03
PC(38:5)_[M+H]1+ / PE(41:5)_[M+H]1+ / PA(43:6)_[M+NH4]1+ 808.5851 0.02 (0.01, 0.02) 2.62e-04*
PG(36:0)_[M-H]1- 777.5649 0.02 (0.01, 0.02) 2.51e-03
PG(34:1)_[M-H]1- 747.518 -0.02 (-0.03, -0.01) 2.66e-03
PG(36:2)_[M-H]1- 773.5337 -0.02 (-0.04, -0.01) 3.97e-04*
PC-O(40:7)_[M+H]1+ / PC-P(40:6)_[M+H]1+ 818.6057 -0.02 (-0.04, -0.01) 5.81e-04*
PI(38:1)_[M-H]1- 891.5967 -0.02 (-0.04, -0.01) 1.69e-03
PC(40:7)_[M+OAc]1- / PS(44:6)_[M-H]1- 890.5916 -0.02 (-0.04, -0.01) 4.99e-04*
PC(37:6)_[M+H]1+ / PE(40:6)_[M+H]1+ / PA(42:7)_[M+NH4]1+ 792.5537 -0.03 (-0.04, -0.01) 2.21e-04*
PC(39:6)_[M+OAc]1- / PS(43:5)_[M-H]1- 878.5916 -0.03 (-0.04, -0.01) 5.31e-04*
PC(38:6)_[M+H]1+ / PE(41:6)_[M+H]1+ 806.5694 -0.03 (-0.04, -0.02) 2.86e-09**
PI(38:6)_[M-H]1- 881.5186 -0.03 (-0.05, -0.02) 1.12e-04*
PC(40:6)_[M+OAc]1- / PS(44:5)_[M-H]1- 892.6072 -0.03 (-0.05, -0.02) 1.05e-05*
PC(40:6)_[M+H]1+ / PE(43:6)_[M+H]1+ 834.6006 -0.03 (-0.05, -0.02) 8.50e-08*
FA(22:6)_[M-H]1- 327.233 -0.04 (-0.06, -0.03) 1.68e-08**
PI(40:6)_[M-H]1- 909.5498 -0.05 (-0.06, -0.03) 1.43e-10***
-.4 -.3 -.2 -.1 0 .1 .2 .3 .4
Beta (95% CI) for ELOVL2 (rs6920155, chr6:11047956)

(h) ***FADS1-FADS2-FADS3***

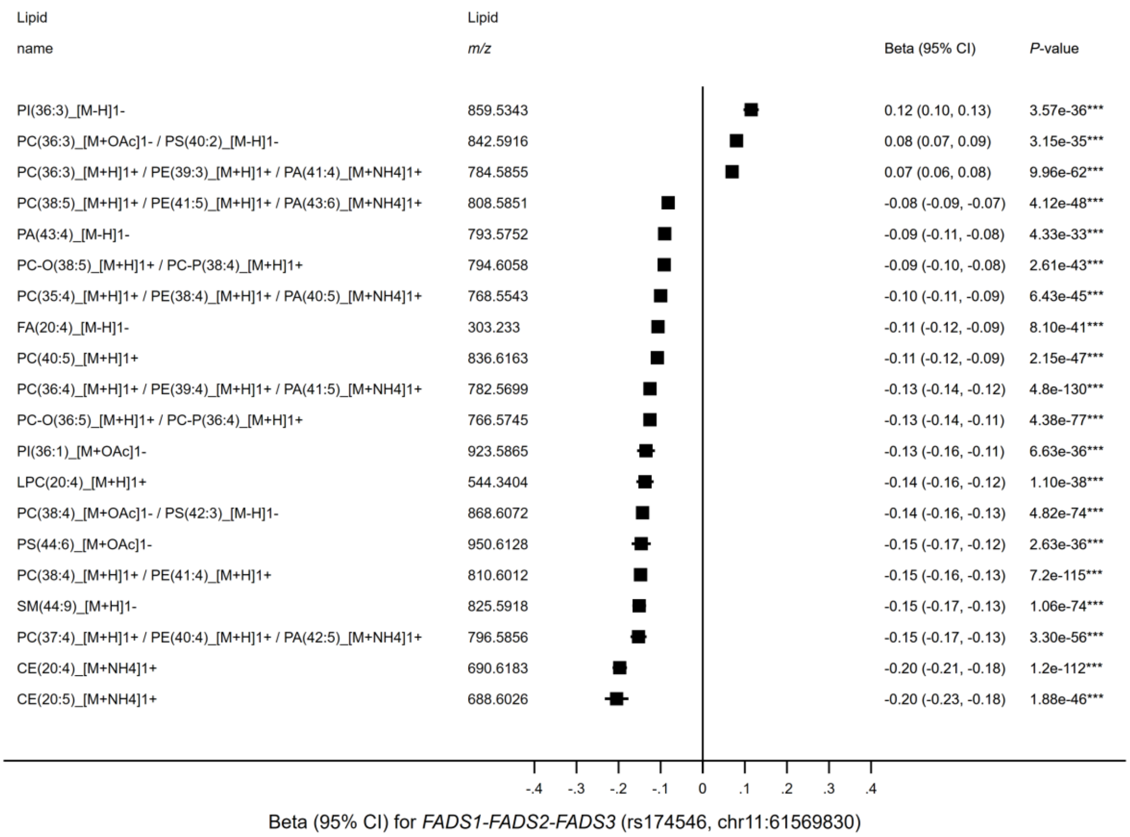

(i) *GAL3ST1*

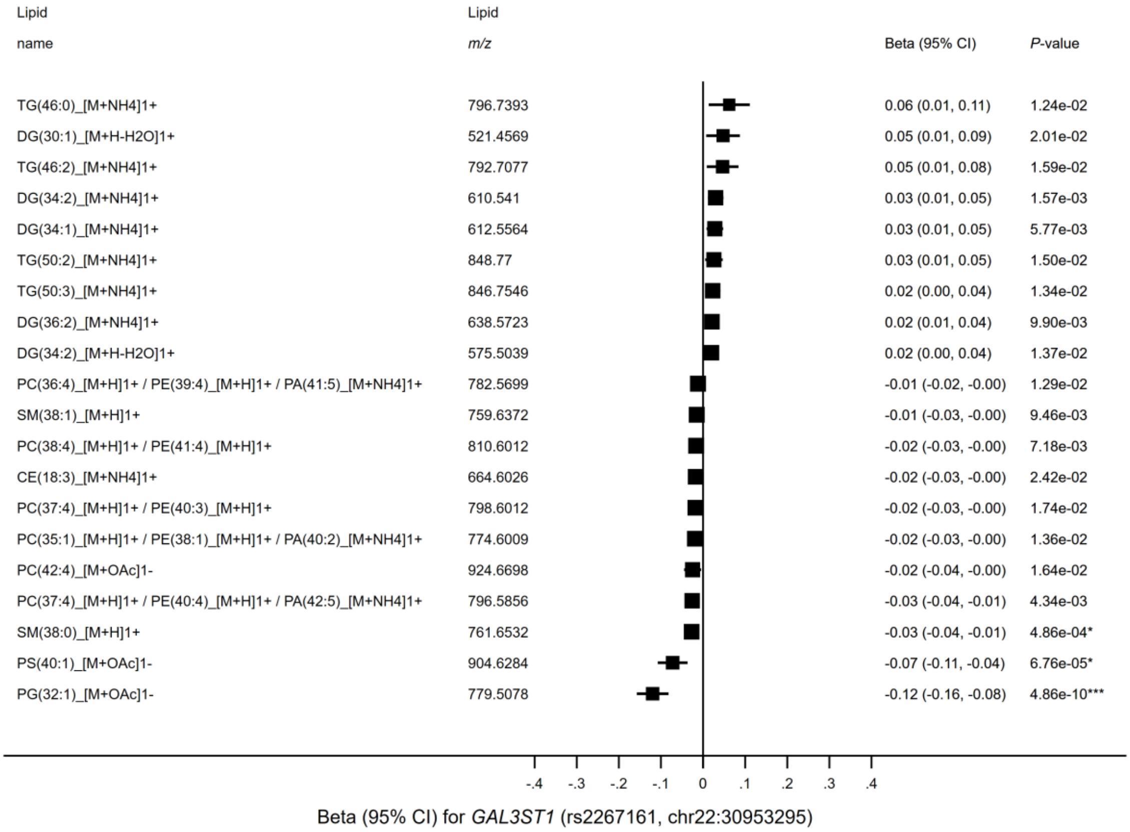
Lipid name
Lipid m/z
Beta (95% CI)
P-value
TG(46:0)_[M+NH4]1+ 796.7393 0.06 (0.01, 0.11) 1.24e-02
DG(30:1)_[M+H-H2O]1+ 521.4569 0.05 (0.01, 0.09) 2.01e-02
TG(46:2)_[M+NH4]1+ 792.7077 0.05 (0.01, 0.08) 1.59e-02
DG(34:2)_[M+NH4]1+ 610.541 0.03 (0.01, 0.05) 1.57e-03
DG(34:1)_[M+NH4]1+ 612.5564 0.03 (0.01, 0.05) 5.77e-03
TG(50:2)_[M+NH4]1+ 848.77 0.03 (0.01, 0.05) 1.50e-02
TG(50:3)_[M+NH4]1+ 846.7546 0.02 (0.00, 0.04) 1.34e-02
DG(36:2)_[M+NH4]1+ 638.5723 0.02 (0.01, 0.04) 9.90e-03
DG(34:2)_[M+H-H2O]1+ 575.5039 0.02 (0.00, 0.04) 1.37e-02
PC(36:4)_[M+H]1+ / PE(39:4)_[M+H]1+ / PA(41:5)_[M+NH4]1+ 782.5699 -0.01 (-0.02, -0.00) 1.29e-02
SM(38:1)_[M+H]1+ 759.6372 -0.01 (-0.03, -0.00) 9.46e-03
PC(38:4)_[M+H]1+ / PE(41:4)_[M+H]1+ 810.6012 -0.02 (-0.03, -0.00) 7.18e-03
CE(18:3)_[M+NH4]1+ 664.6026 -0.02 (-0.03, -0.00) 2.42e-02
PC(37:4)_[M+H]1+ / PE(40:3)_[M+H]1+ 798.6012 -0.02 (-0.03, -0.00) 1.74e-02
PC(35:1)_[M+H]1+ / PE(38:1)_[M+H]1+ / PA(40:2)_[M+NH4]1+ 774.6009 -0.02 (-0.03, -0.00) 1.36e-02
PC(42:4)_[M+OAc]1- 924.6698 -0.02 (-0.04, -0.00) 1.64e-02
PC(37:4)_[M+H]1+ / PE(40:4)_[M+H]1+ / PA(42:5)_[M+NH4]1+ 796.5856 -0.03 (-0.04, -0.01) 4.34e-03
SM(38:0)_[M+H]1+ 761.6532 -0.03 (-0.04, -0.01) 4.86e-04*
PS(40:1)_[M+OAc]1- 904.6284 -0.07 (-0.11, -0.04) 6.76e-05*
PG(32:1)_[M+OAc]1- 779.5078 -0.12 (-0.16, -0.08) 4.86e-10***
-.4 -.3 -.2 -.1 0 .1 .2 .3 .4
Beta (95% CI) for GAL3ST1 (rs2267161, chr22:30953295)

(j) *GCKR*

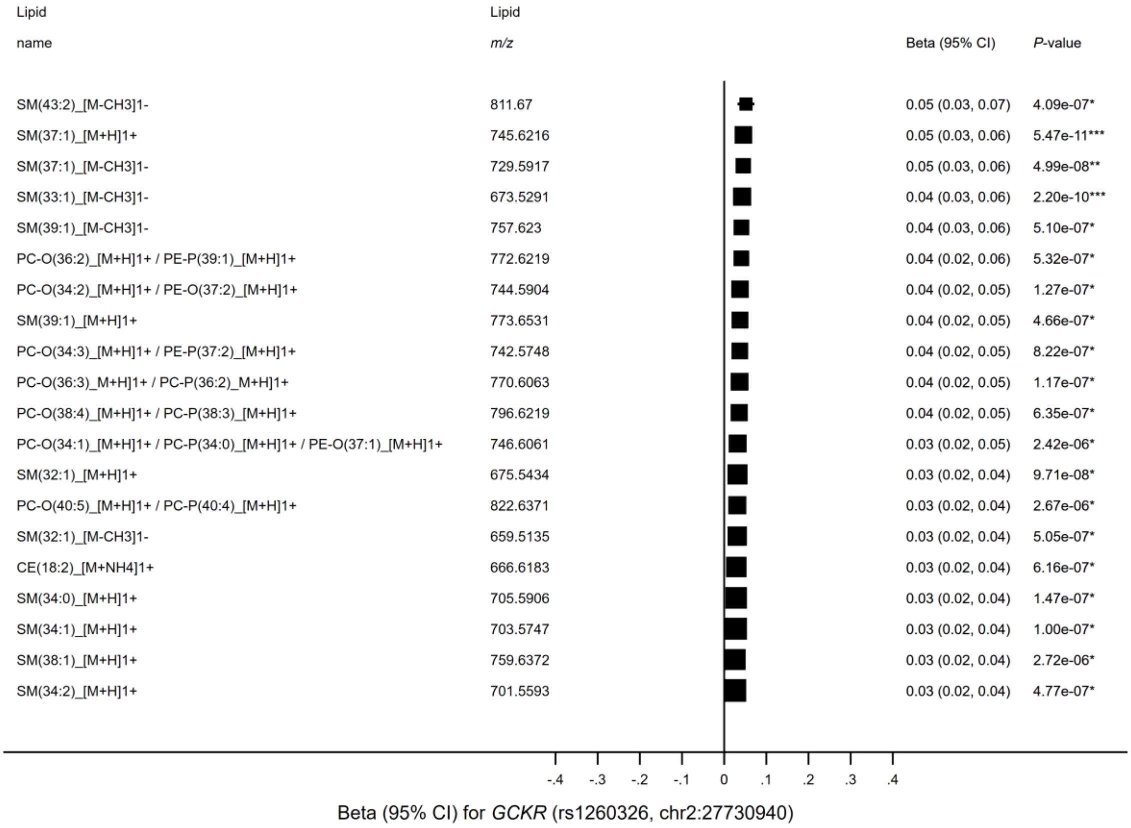
Lipid name
Lipid m/z
Beta (95% CI)
P-value
SM(43:2)_[M-CH3]1- 811.67 0.05 (0.03, 0.07) 4.09e-07*
SM(37:1)_[M+H]1+ 745.6216 0.05 (0.03, 0.06) 5.47e-11***
SM(37:1)_[M-CH3]1- 729.5917 0.05 (0.03, 0.06) 4.99e-08**
SM(33:1)_[M-CH3]1- 673.5291 0.04 (0.03, 0.06) 2.20e-10***
SM(39:1)_[M-CH3]1- 757.623 0.04 (0.03, 0.06) 5.10e-07*
PC-O(36:2)_[M+H]1+ / PE-P(39:1)_[M+H]1+ 772.6219 0.04 (0.02, 0.06) 5.32e-07*
PC-O(34:2)_[M+H]1+ / PE-O(37:2)_[M+H]1+ 744.5904 0.04 (0.02, 0.05) 1.27e-07*
SM(39:1)_[M+H]1+ 773.6531 0.04 (0.02, 0.05) 4.66e-07*
PC-O(34:3)_[M+H]1+ / PE-P(37:2)_[M+H]1+ 742.5748 0.04 (0.02, 0.05) 8.22e-07*
PC-O(36:3)_M+H]1+ / PC-P(36:2)_M+H]1+ 770.6063 0.04 (0.02, 0.05) 1.17e-07*
PC-O(38:4)_[M+H]1+ / PC-P(38:3)_[M+H]1+ 796.6219 0.04 (0.02, 0.05) 6.35e-07*
PC-O(34:1)_[M+H]1+ / PC-P(34:0)_[M+H]1+ / PE-O(37:1)_[M+H]1+ 746.6061 0.03 (0.02, 0.05) 2.42e-06*
SM(32:1)_[M+H]1+ 675.5434 0.03 (0.02, 0.04) 9.71e-08*
PC-O(40:5)_[M+H]1+ / PC-P(40:4)_[M+H]1+ 822.6371 0.03 (0.02, 0.04) 2.67e-06*
SM(32:1)_[M-CH3]1- 659.5135 0.03 (0.02, 0.04) 5.05e-07*
CE(18:2)_[M+NH4]1+ 666.6183 0.03 (0.02, 0.04) 6.16e-07*
SM(34:0)_[M+H]1+ 705.5906 0.03 (0.02, 0.04) 1.47e-07*
SM(34:1)_[M+H]1+ 703.5747 0.03 (0.02, 0.04) 1.00e-07*
SM(38:1)_[M+H]1+ 759.6372 0.03 (0.02, 0.04) 2.72e-06*
SM(34:2)_[M+H]1+ 701.5593 0.03 (0.02, 0.04) 4.77e-07*
-.4 -.3 -.2 -.1 0 .1 .2 .3 .4
Beta (95% CI) for GCKR (rs1260326, chr2:27730940)

(k) *LIPC*

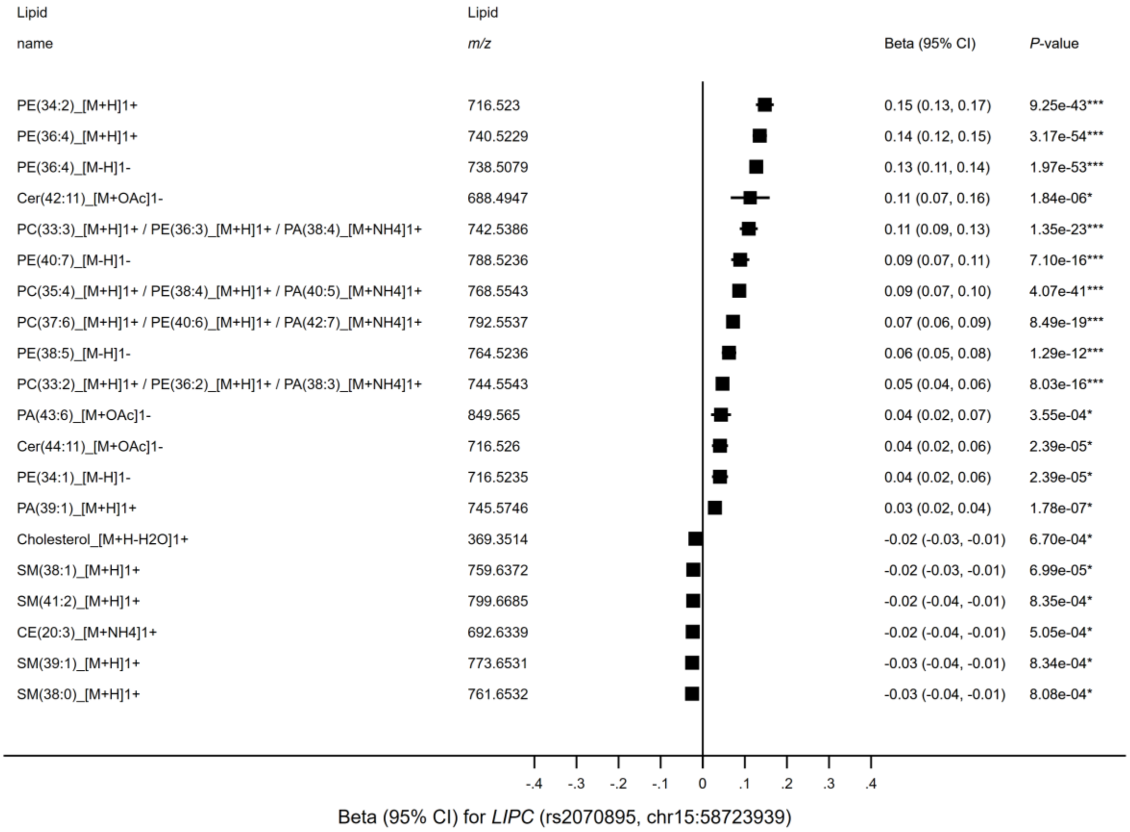
Lipid name
Lipid m/z
Beta (95% CI)
P-value
PE(34:2)_[M+H]1+ 716.523 0.15 (0.13, 0.17) 9.25e-43***
PE(36:4)_[M+H]1+ 740.5229 0.14 (0.12, 0.15) 3.17e-54***
PE(36:4)_[M-H]1- 738.5079 0.13 (0.11, 0.14) 1.97e-53***
Cer(42:11)_[M+OAc]1- 688.4947 0.11 (0.07, 0.16) 1.84e-06*
PC(33:3)_[M+H]1+ / PE(36:3)_[M+H]1+ / PA(38:4)_[M+NH4]1+ 742.5386 0.11 (0.09, 0.13) 1.35e-23***
PE(40:7)_[M-H]1- 788.5236 0.09 (0.07, 0.11) 7.10e-16***
PC(35:4)_[M+H]1+ / PE(38:4)_[M+H]1+ / PA(40:5)_[M+NH4]1+ 768.5543 0.09 (0.07, 0.10) 4.07e-41***
PC(37:6)_[M+H]1+ / PE(40:6)_[M+H]1+ / PA(42:7)_[M+NH4]1+ 792.5537 0.07 (0.06, 0.09) 8.49e-19***
PE(38:5)_[M-H]1- 764.5236 0.06 (0.05, 0.08) 1.29e-12***
PC(33:2)_[M+H]1+ / PE(36:2)_[M+H]1+ / PA(38:3)_[M+NH4]1+ 744.5543 0.05 (0.04, 0.06) 8.03e-16***
PA(43:6)_[M+OAc]1- 849.565 0.04 (0.02, 0.07) 3.55e-04*
Cer(44:11)_[M+OAc]1- 716.526 0.04 (0.02, 0.06) 2.39e-05*
PE(34:1)_[M-H]1- 716.5235 0.04 (0.02, 0.06) 2.39e-05*
PA(39:1)_[M+H]1+ 745.5746 0.03 (0.02, 0.04) 1.78e-07*
Cholesterol_[M+H-H2O]1+ 369.3514 -0.02 (-0.03, -0.01) 6.70e-04*
SM(38:1)_[M+H]1+ 759.6372 -0.02 (-0.03, -0.01) 6.99e-05*
SM(41:2)_[M+H]1+ 799.6685 -0.02 (-0.04, -0.01) 8.35e-04*
CE(20:3)_[M+NH4]1+ 692.6339 -0.02 (-0.04, -0.01) 5.05e-04*
SM(39:1)_[M+H]1+ 773.6531 -0.03 (-0.04, -0.01) 8.34e-04*
SM(38:0)_[M+H]1+ 761.6532 -0.03 (-0.04, -0.01) 8.08e-04*
-.4 -.3 -.2 -.1 0 .1 .2 .3 .4
Beta (95% CI) for LIPC (rs2070895, chr15:58723939)

(l) *LPL*

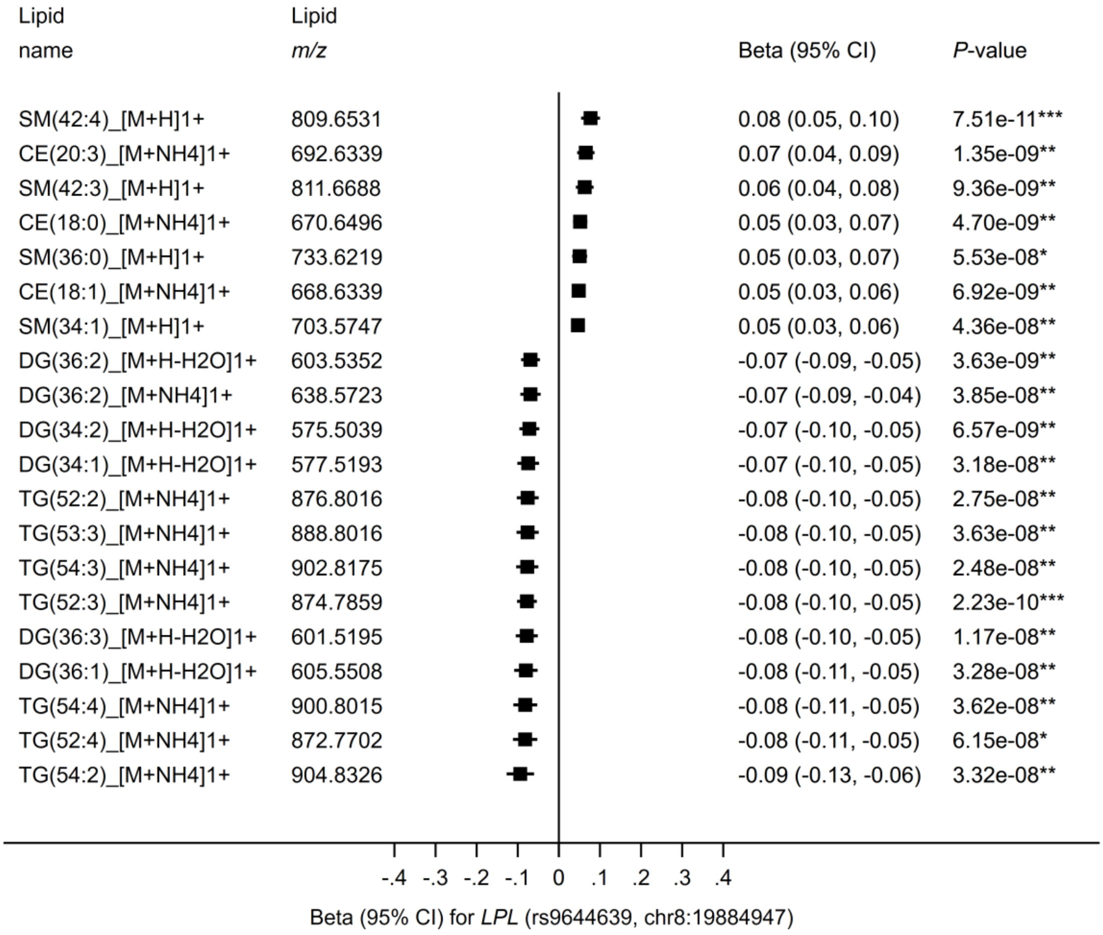

Lipid name
Lipid m/z
Beta (95% CI)
P-value
SM(42:4)_[M+H]1+ 809.6531 0.08 (0.05, 0.10) 7.51e-11***
CE(20:3)_[M+NH4]1+ 692.6339 0.07 (0.04, 0.09) 1.35e-09**
SM(42:3)_[M+H]1+ 811.6688 0.06 (0.04, 0.08) 9.36e-09**
CE(18:0)_[M+NH4]1+ 670.6496 0.05 (0.03, 0.07) 4.70e-09**
SM(36:0)_[M+H]1+ 733.6219 0.05 (0.03, 0.07) 5.53e-08*
CE(18:1)_[M+NH4]1+ 668.6339 0.05 (0.03, 0.06) 6.92e-09**
SM(34:1)_[M+H]1+ 703.5747 0.05 (0.03, 0.06) 4.36e-08**
DG(36:2)_[M+H-H2O]1+ 603.5352 -0.07 (-0.09, -0.05) 3.63e-09**
DG(36:2)_[M+NH4]1+ 638.5723 -0.07 (-0.09, -0.04) 3.85e-08**
DG(34:2)_[M+H-H2O]1+ 575.5039 -0.07 (-0.10, -0.05) 6.57e-09**
DG(34:1)_[M+H-H2O]1+ 577.5193 -0.07 (-0.10, -0.05) 3.18e-08**
TG(52:2)_[M+NH4]1+ 876.8016 -0.08 (-0.10, -0.05) 2.75e-08**
TG(53:3)_[M+NH4]1+ 888.8016 -0.08 (-0.10, -0.05) 3.63e-08**
TG(54:3)_[M+NH4]1+ 902.8175 -0.08 (-0.10, -0.05) 2.48e-08**
TG(52:3)_[M+NH4]1+ 874.7859 -0.08 (-0.10, -0.05) 2.23e-10***
DG(36:3)_[M+H-H2O]1+ 601.5195 -0.08 (-0.10, -0.05) 1.17e-08**
DG(36:1)_[M+H-H2O]1+ 605.5508 -0.08 (-0.11, -0.05) 3.28e-08**
TG(54:4)_[M+NH4]1+ 900.8015 -0.08 (-0.11, -0.05) 3.62e-08**
TG(52:4)_[M+NH4]1+ 872.7702 -0.08 (-0.11, -0.05) 6.15e-08*
TG(54:2)_[M+NH4]1+ 904.8326 -0.09 (-0.13, -0.06) 3.32e-08**
-.4 -.3 -.2 -.1 0 .1 .2 .3 .4
Beta (95% CI) for LPL (rs9644639, chr8:19884947)

(m) ***MBOAT7***

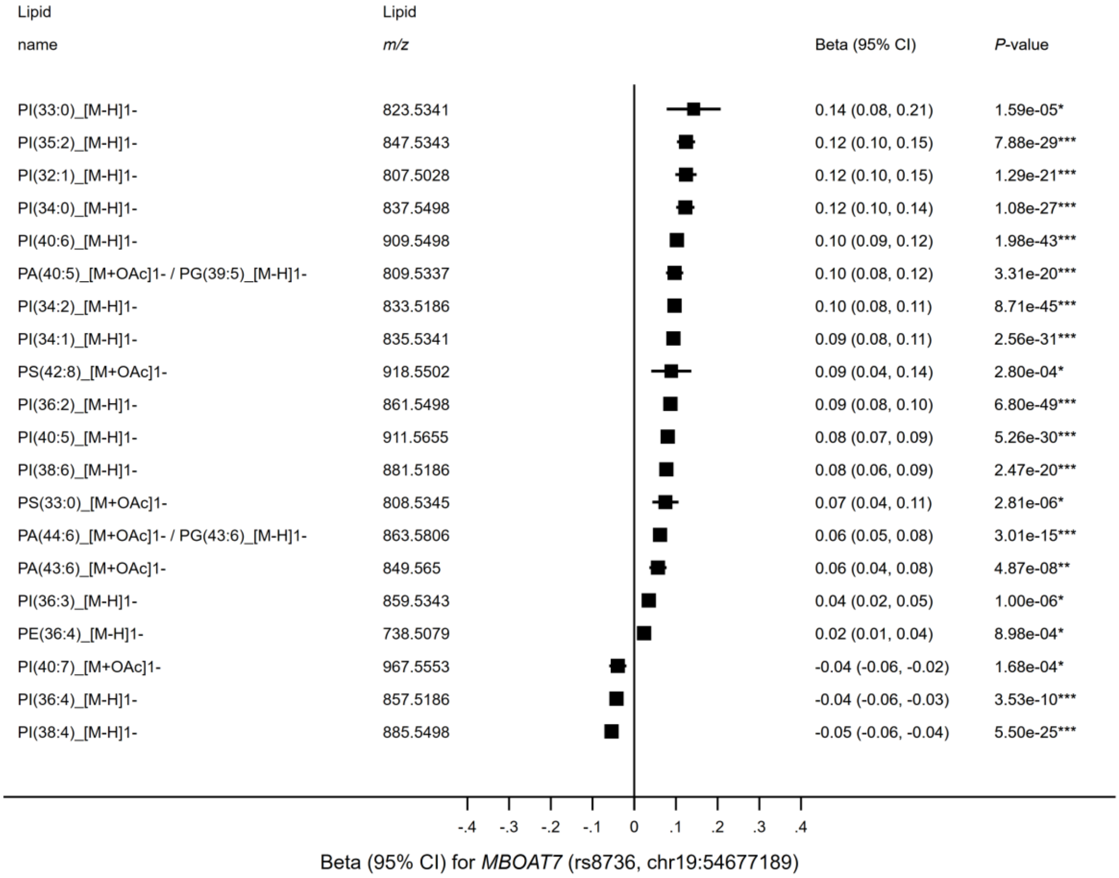
Lipid name
Lipid m/z
Beta (95% CI)
P-value
PI(33:0)_[M-H]1- 823.5341 0.14 (0.08, 0.21) 1.59e-05*
PI(35:2)_[M-H]1- 847.5343 0.12 (0.10, 0.15) 7.88e-29***
PI(32:1)_[M-H]1- 807.5028 0.12 (0.10, 0.15) 1.29e-21***
PI(34:0)_[M-H]1- 837.5498 0.12 (0.10, 0.14) 1.08e-27***
PI(40:6)_[M-H]1- 909.5498 0.10 (0.09, 0.12) 1.98e-43***
PA(40:5)_[M+OAc]1- / PG(39:5)_[M-H]1- 809.5337 0.10 (0.08, 0.12) 3.31e-20***
PI(34:2)_[M-H]1- 833.5186 0.10 (0.08, 0.11) 8.71e-45***
PI(34:1)_[M-H]1- 835.5341 0.09 (0.08, 0.11) 2.56e-31***
PS(42:8)_[M+OAc]1- 918.5502 0.09 (0.04, 0.14) 2.80e-04*
PI(36:2)_[M-H]1- 861.5498 0.09 (0.08, 0.10) 6.80e-49***
PI(40:5)_[M-H]1- 911.5655 0.08 (0.07, 0.09) 5.26e-30***
PI(38:6)_[M-H]1- 881.5186 0.08 (0.06, 0.09) 2.47e-20***
PS(33:0)_[M+OAc]1- 808.5345 0.07 (0.04, 0.11) 2.81e-06*
PA(44:6)_[M+OAc]1- / PG(43:6)_[M-H]1- 863.5806 0.06 (0.05, 0.08) 3.01e-15***
PA(43:6)_[M+OAc]1- 849.565 0.06 (0.04, 0.08) 4.87e-08**
PI(36:3)_[M-H]1- 859.5343 0.04 (0.02, 0.05) 1.00e-06*
PE(36:4)_[M-H]1- 738.5079 0.02 (0.01, 0.04) 8.98e-04*
PI(40:7)_[M+OAc]1- 967.5553 -0.04 (-0.06, -0.02) 1.68e-04*
PI(36:4)_[M-H]1- 857.5186 -0.04 (-0.06, -0.03) 3.53e-10***
PI(38:4)_[M-H]1- 885.5498 -0.05 (-0.06, -0.04) 5.50e-25***
-.4 -.3 -.2 -.1 0 .1 .2 .3 .4
Beta (95% CI) for MBOAT7 (rs8736, chr19:54677189)

(n) ***MLXIPL***

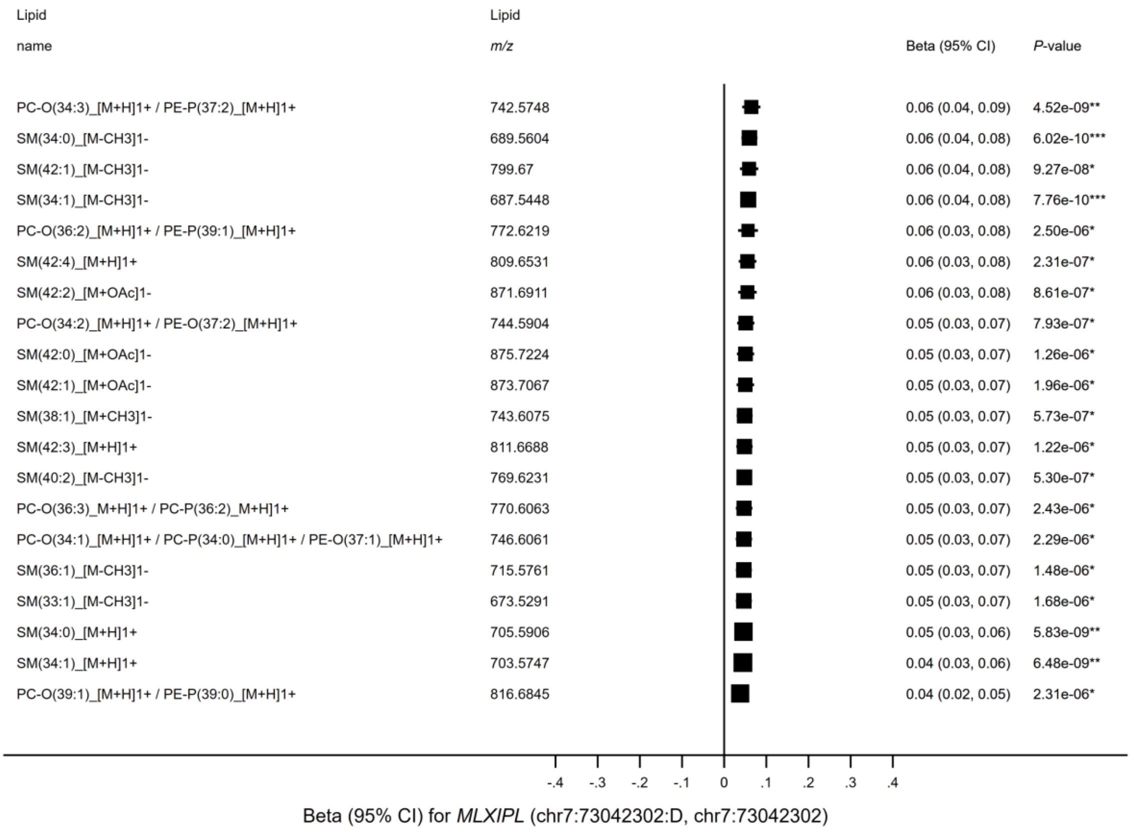
Lipid name
Lipid m/z
Beta (95% CI)
P-value
PC-O(34:3)_[M+H]1+ / PE-P(37:2)_[M+H]1+ 742.5748 0.06 (0.04, 0.09) 4.52e-09**
SM(34:0)_[M-CH3]1- 689.5604 0.06 (0.04, 0.08) 6.02e-10***
SM(42:1)_[M-CH3]1- 799.67 0.06 (0.04, 0.08) 9.27e-08*
SM(34:1)_[M-CH3]1- 687.5448 0.06 (0.04, 0.08) 7.76e-10***
PC-O(36:2)_[M+H]1+ / PE-P(39:1)_[M+H]1+ 772.6219 0.06 (0.03, 0.08) 2.50e-06*
SM(42:4)_[M+H]1+ 809.6531 0.06 (0.03, 0.08) 2.31e-07*
SM(42:2)_[M+OAc]1- 871.6911 0.06 (0.03, 0.08) 8.61e-07*
PC-O(34:2)_[M+H]1+ / PE-O(37:2)_[M+H]1+ 744.5904 0.05 (0.03, 0.07) 7.93e-07*
SM(42:0)_[M+OAc]1- 875.7224 0.05 (0.03, 0.07) 1.26e-06*
SM(42:1)_[M+OAc]1- 873.7067 0.05 (0.03, 0.07) 1.96e-06*
SM(38:1)_[M+CH3]1- 743.6075 0.05 (0.03, 0.07) 5.73e-07*
SM(42:3)_[M+H]1+ 811.6688 0.05 (0.03, 0.07) 1.22e-06*
SM(40:2)_[M-CH3]1- 769.6231 0.05 (0.03, 0.07) 5.30e-07*
PC-O(36:3)_M+H]1+ / PC-P(36:2)_M+H]1+ 770.6063 0.05 (0.03, 0.07) 2.43e-06*
PC-O(34:1)_[M+H]1+ / PC-P(34:0)_[M+H]1+ / PE-O(37:1)_[M+H]1+ 746.6061 0.05 (0.03, 0.07) 2.29e-06*
SM(36:1)_[M-CH3]1- 715.5761 0.05 (0.03, 0.07) 1.48e-06*
SM(33:1)_[M-CH3]1- 673.5291 0.05 (0.03, 0.07) 1.68e-06*
SM(34:0)_[M+H]1+ 705.5906 0.05 (0.03, 0.06) 5.83e-09**
SM(34:1)_[M+H]1+ 703.5747 0.04 (0.03, 0.06) 6.48e-09**
PC-O(39:1)_[M+H]1+ / PE-P(39:0)_[M+H]1+ 816.6845 0.04 (0.02, 0.05) 2.31e-06*
-.4 -.3 -.2 -.1 0 .1 .2 .3 .4
Beta (95% CI) for MLXIPL (chr7:73042302:D, chr7:73042302)

(o) ***PAQR9***

Lipid name	Lipid *m/z*	Beta (95% CI)	*P*-value
PA(44:6)_[M+OAc]1- / PG(43:6)_[M-H]1-	863.5806	0.08 (0.05, 0.10)	3.71e-10***
PI(34:0)_[M-H]1-	837.5498	0.06 (0.03, 0.10)	6.81e-04*
PI(34:1)_[M-H]1-	835.5341	0.05 (0.02, 0.07)	2.59e-04*
CE(18:0)_[M+NH4]1+	670.6496	0.02 (0.01, 0.04)	3.07e-03
CE(18:1)_[M+NH4]1+	668.6339	0.02 (0.01, 0.04)	3.36e-03
CE(16:0)_[M+NH4]1+	642.6183	0.01 (0.00, 0.03)	4.60e-02
PC(36:3)_[M+OAc]1- / PS(40:2)_[M-H]1-	842.5916	-0.02 (-0.03, -0.00)	3.80e-02
PC(34:3)_[M+H]1+ / PE(37:3)_[M+H]1+ / PA(39:4)_[M+NH4]1+	756.5541	-0.02 (-0.04, -0.00)	3.18e-02
PG(35:3)_[M-H]1-	757.5024	-0.02 (-0.04, 0.00)	5.23e-02
PA(43:6)_[M-H]1-	789.5439	-0.02 (-0.04, -0.00)	5.09e-02
PA(45:6)_[M-H]1-	817.5752	-0.02 (-0.04, -0.00)	1.58e-02
PI(36:4)_[M-H]1-	857.5186	-0.02 (-0.04, -0.00)	2.62e-02
PE(38:5)_[M-H]1-	764.5236	-0.03 (-0.05, -0.00)	2.04e-02
DG(34:3)_[M+H-H2O]1+	573.488	-0.03 (-0.06, -0.00)	2.90e-02
PC(32:1)_[M+OAc]1-	790.5602	-0.03 (-0.06, -0.00)	4.14e-02
PE(36:4)_[M-H]1-	738.5079	-0.03 (-0.06, -0.01)	3.42e-03
PE(40:7)_[M-H]1-	788.5236	-0.03 (-0.06, -0.01)	2.16e-02
PA(40:5)_[M+OAc]1- / PG(39:5)_[M-H]1-	809.5337	-0.04 (-0.07, -0.00)	3.19e-02
PS(38:3) _[M+OAc]1-	872.5659	-0.04 (-0.08, -0.01)	2.46e-02
PS(40:5) _[M+OAc]1-	896.5659	-0.05 (-0.08, -0.01)	8.69e-03

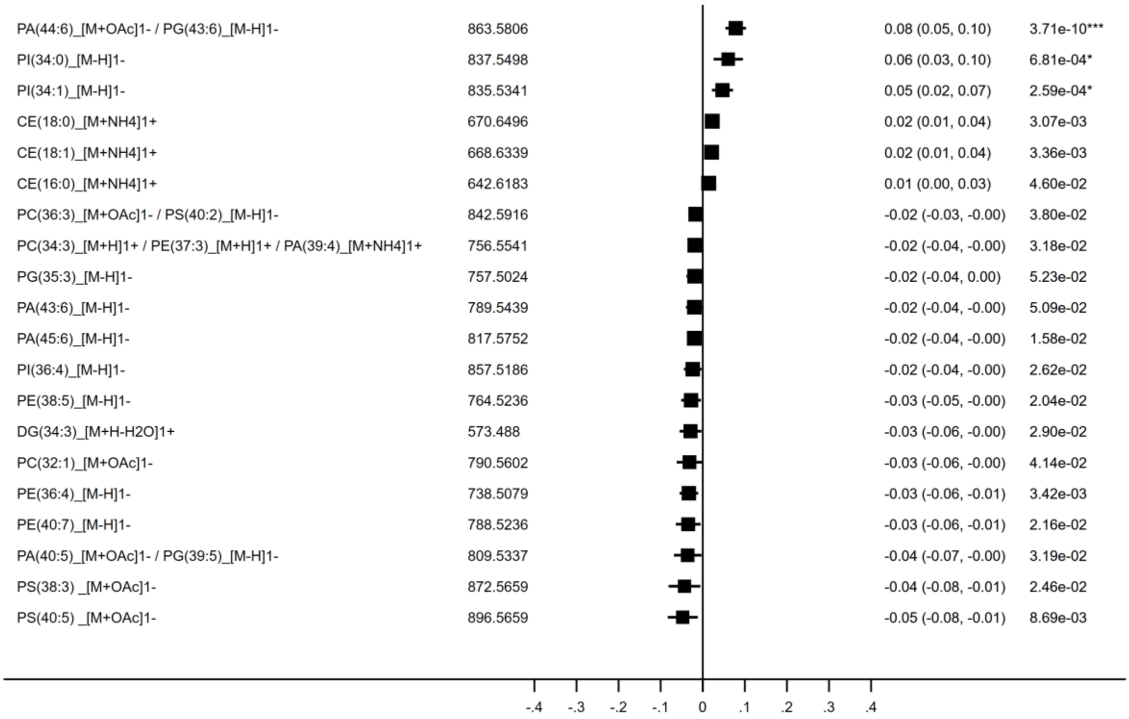

Beta (95% CI) for *PAQR9* (chr3:142660022:D, chr3:142660022)

(p) *PCTP*

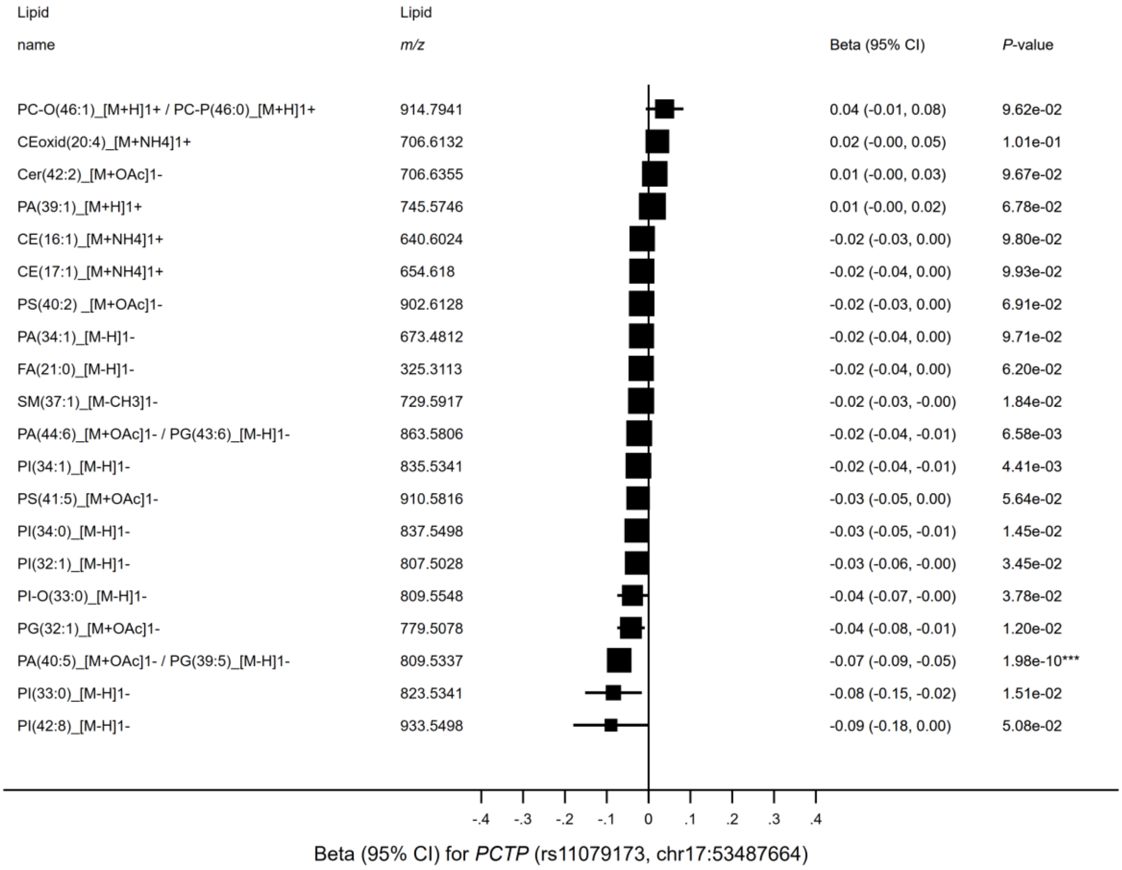

Lipid name
Lipid m/z
Beta (95% CI)
P-value
PC-O(46:1)_[M+H]1+ / PC-P(46:0)_[M+H]1+ 914.7941 0.04 (-0.01, 0.08) 9.62e-02
CEoxid(20:4)_[M+NH4]1+ 706.6132 0.02 (-0.00, 0.05) 1.01e-01
Cer(42:2)_[M+OAc]1- 706.6355 0.01 (-0.00, 0.03) 9.67e-02
PA(39:1)_[M+H]1+ 745.5746 0.01 (-0.00, 0.02) 6.78e-02
CE(16:1)_[M+NH4]1+ 640.6024 -0.02 (-0.03, 0.00) 9.80e-02
CE(17:1)_[M+NH4]1+ 654.618 -0.02 (-0.04, 0.00) 9.93e-02
PS(40:2) _[M+OAc]1- 902.6128 -0.02 (-0.03, 0.00) 6.91e-02
PA(34:1)_[M-H]1- 673.4812 -0.02 (-0.04, 0.00) 9.71e-02
FA(21:0)_[M-H]1- 325.3113 -0.02 (-0.04, 0.00) 6.20e-02
SM(37:1)_[M-CH3]1- 729.5917 -0.02 (-0.03, -0.00) 1.84e-02
PA(44:6)_[M+OAc]1- / PG(43:6)_[M-H]1- 863.5806 -0.02 (-0.04, -0.01) 6.58e-03
PI(34:1)_[M-H]1- 835.5341 -0.02 (-0.04, -0.01) 4.41e-03
PS(41:5)_[M+OAc]1- 910.5816 -0.03 (-0.05, 0.00) 5.64e-02
PI(34:0)_[M-H]1- 837.5498 -0.03 (-0.05, -0.01) 1.45e-02
PI(32:1)_[M-H]1- 807.5028 -0.03 (-0.06, -0.00) 3.45e-02
PI-O(33:0)_[M-H]1- 809.5548 -0.04 (-0.07, -0.00) 3.78e-02
PG(32:1)_[M+OAc]1- 779.5078 -0.04 (-0.08, -0.01) 1.20e-02
PA(40:5)_[M+OAc]1- / PG(39:5)_[M-H]1- 809.5337 -0.07 (-0.09, -0.05) 1.98e-10***
PI(33:0)_[M-H]1- 823.5341 -0.08 (-0.15, -0.02) 1.51e-02
PI(42:8)_[M-H]1- 933.5498 -0.09 (-0.18, 0.00) 5.08e-02
-.4 -.3 -.2 -.1 0 .1 .2 .3 .4
Beta (95% CI) for PCTP (rs11079173, chr17:53487664)

(q) *PIGH-TMEM229B*

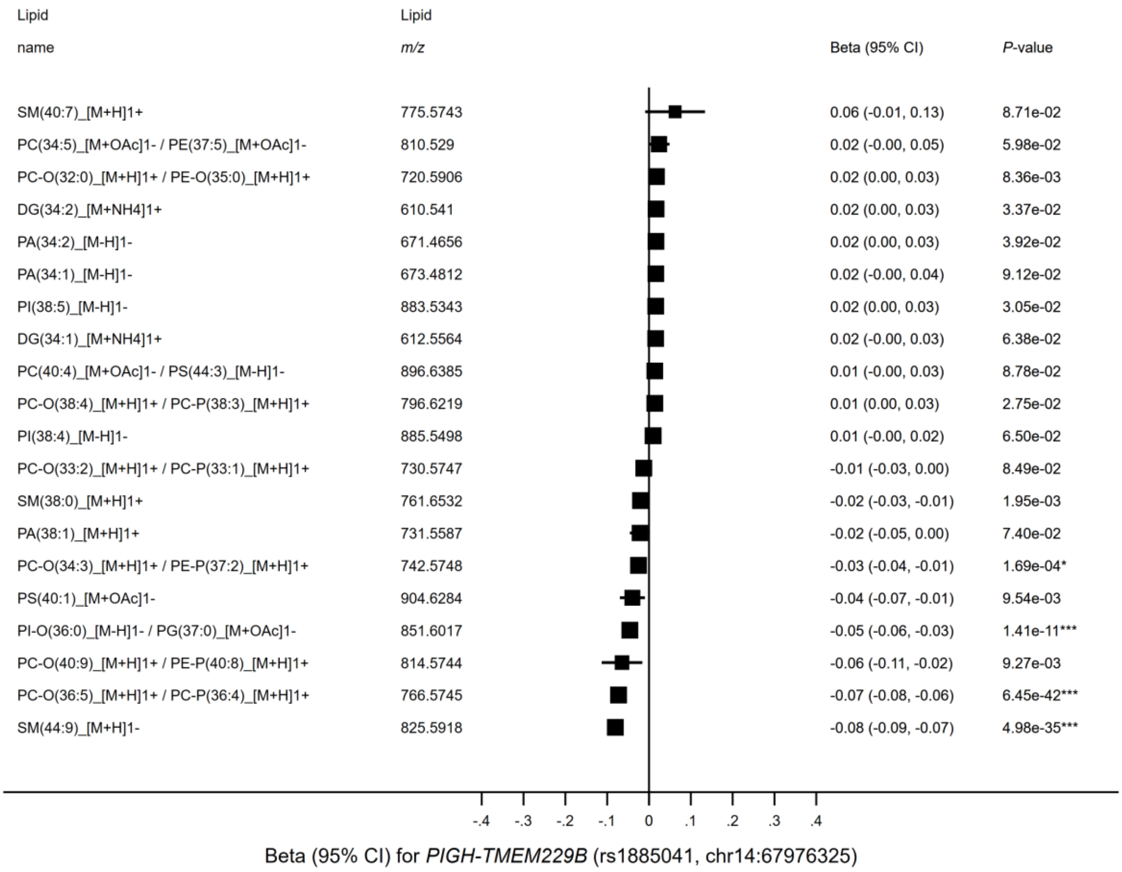

(r) ***PLA2G10-NTAN1-NPIPA5***

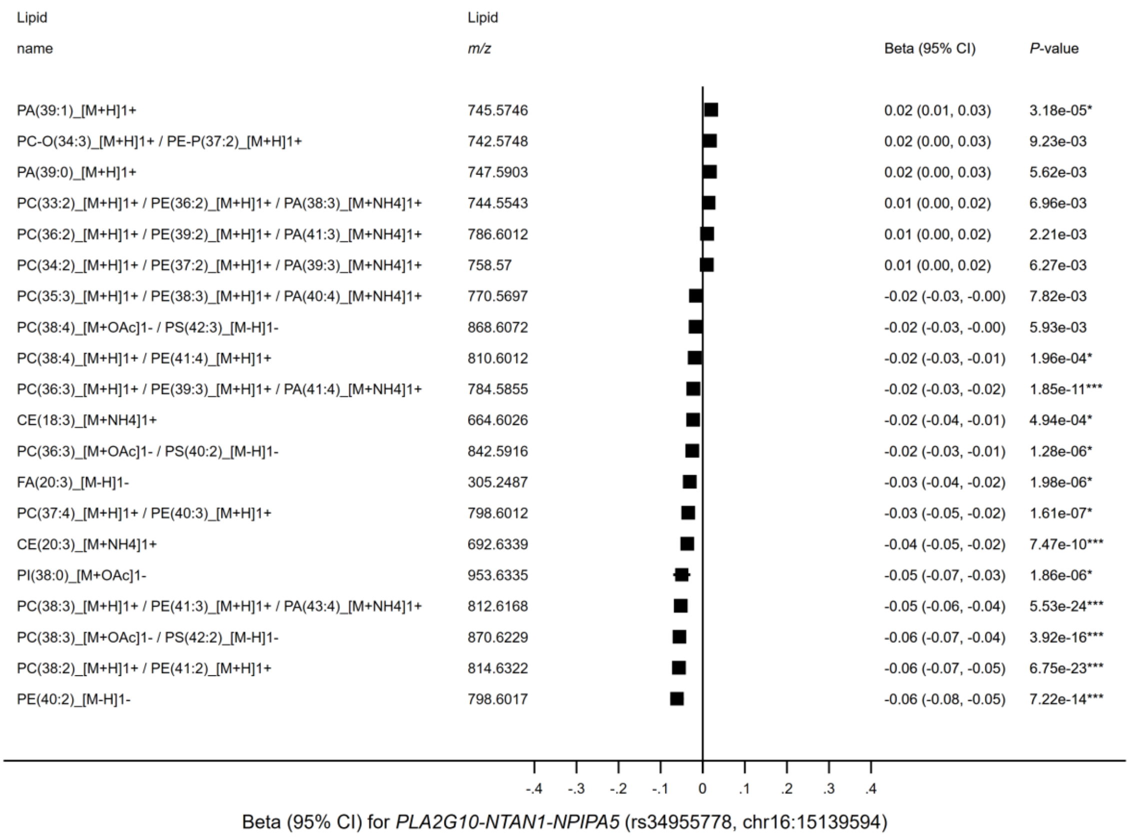

(s) *PNPLA3*

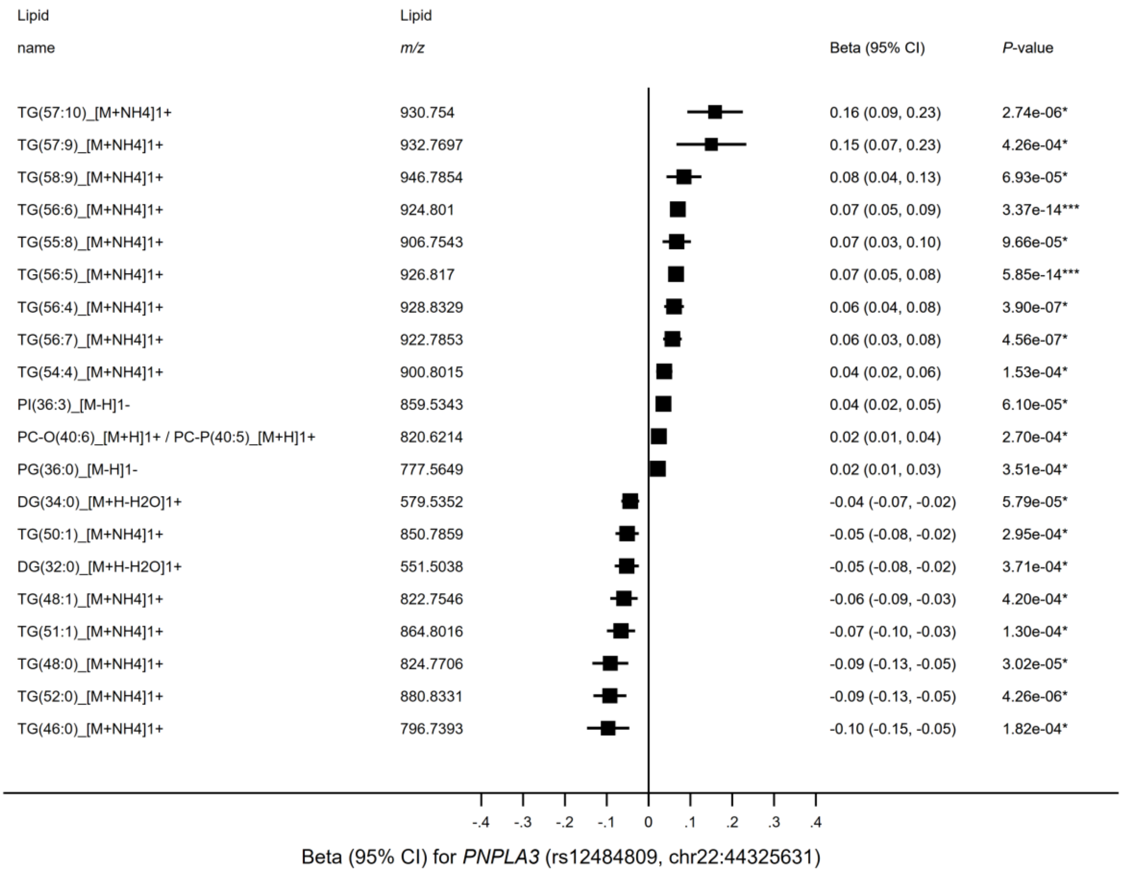
Lipid name
Lipid m/z
Beta (95% CI)
P-value
TG(57:10)_[M+NH4]1+ 930.754 0.16 (0.09, 0.23) 2.74e-06*
TG(57:9)_[M+NH4]1+ 932.7697 0.15 (0.07, 0.23) 4.26e-04*
TG(58:9)_[M+NH4]1+ 946.7854 0.08 (0.04, 0.13) 6.93e-05*
TG(56:6)_[M+NH4]1+ 924.801 0.07 (0.05, 0.09) 3.37e-14***
TG(55:8)_[M+NH4]1+ 906.7543 0.07 (0.03, 0.10) 9.66e-05*
TG(56:5)_[M+NH4]1+ 926.817 0.07 (0.05, 0.08) 5.85e-14***
TG(56:4)_[M+NH4]1+ 928.8329 0.06 (0.04, 0.08) 3.90e-07*
TG(56:7)_[M+NH4]1+ 922.7853 0.06 (0.03, 0.08) 4.56e-07*
TG(54:4)_[M+NH4]1+ 900.8015 0.04 (0.02, 0.06) 1.53e-04*
PI(36:3)_[M-H]1- 859.5343 0.04 (0.02, 0.05) 6.10e-05*
PC-O(40:6)_[M+H]1+ / PC-P(40:5)_[M+H]1+ 820.6214 0.02 (0.01, 0.04) 2.70e-04*
PG(36:0)_[M-H]1- 777.5649 0.02 (0.01, 0.03) 3.51e-04*
DG(34:0)_[M+H-H2O]1+ 579.5352 -0.04 (-0.07, -0.02) 5.79e-05*
TG(50:1)_[M+NH4]1+ 850.7859 -0.05 (-0.08, -0.02) 2.95e-04*
DG(32:0)_[M+H-H2O]1+ 551.5038 -0.05 (-0.08, -0.02) 3.71e-04*
TG(48:1)_[M+NH4]1+ 822.7546 -0.06 (-0.09, -0.03) 4.20e-04*
TG(51:1)_[M+NH4]1+ 864.8016 -0.07 (-0.10, -0.03) 1.30e-04*
TG(48:0)_[M+NH4]1+ 824.7706 -0.09 (-0.13, -0.05) 3.02e-05*
TG(52:0)_[M+NH4]1+ 880.8331 -0.09 (-0.13, -0.05) 4.26e-06*
TG(46:0)_[M+NH4]1+ 796.7393 -0.10 (-0.15, -0.05) 1.82e-04*
-.4 -.3 -.2 -.1 0 .1 .2 .3 .4
Beta (95% CI) for PNPLA3 (rs12484809, chr22:44325631)

(t) ***SCD***

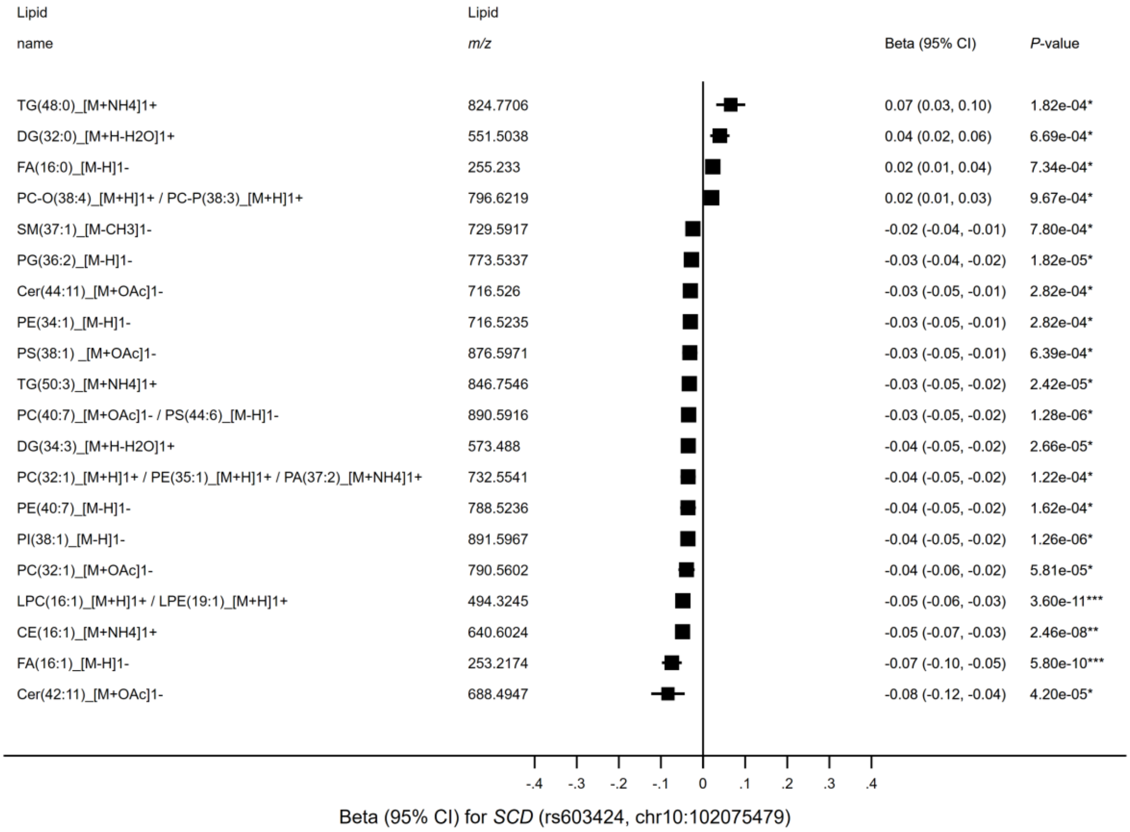

Lipid name
Lipid m/z
Beta (95% CI)
P-value
TG(48:0)_[M+NH4]1+ 824.7706 0.07 (0.03, 0.10) 1.82e-04*
DG(32:0)_[M+H-H2O]1+ 551.5038 0.04 (0.02, 0.06) 6.69e-04*
FA(16:0)_[M-H]1- 255.233 0.02 (0.01, 0.04) 7.34e-04*
PC-O(38:4)_[M+H]1+ / PC-P(38:3)_[M+H]1+ 796.6219 0.02 (0.01, 0.03) 9.67e-04*
SM(37:1)_[M-CH3]1- 729.5917 -0.02 (-0.04, -0.01) 7.80e-04*
PG(36:2)_[M-H]1- 773.5337 -0.03 (-0.04, -0.02) 1.82e-05*
Cer(44:11)_[M+OAc]1- 716.526 -0.03 (-0.05, -0.01) 2.82e-04*
PE(34:1)_[M-H]1- 716.5235 -0.03 (-0.05, -0.01) 2.82e-04*
PS(38:1) _[M+OAc]1- 876.5971 -0.03 (-0.05, -0.01) 6.39e-04*
TG(50:3)_[M+NH4]1+ 846.7546 -0.03 (-0.05, -0.02) 2.42e-05*
PC(40:7)_[M+OAc]1- / PS(44:6)_[M-H]1- 890.5916 -0.03 (-0.05, -0.02) 1.28e-06*
DG(34:3)_[M+H-H2O]1+ 573.488 -0.04 (-0.05, -0.02) 2.66e-05*
PC(32:1)_[M+H]1+ / PE(35:1)_[M+H]1+ / PA(37:2)_[M+NH4]1+ 732.5541 -0.04 (-0.05, -0.02) 1.22e-04*
PE(40:7)_[M-H]1- 788.5236 -0.04 (-0.05, -0.02) 1.62e-04*
PI(38:1)_[M-H]1- 891.5967 -0.04 (-0.05, -0.02) 1.26e-06*
PC(32:1)_[M+OAc]1- 790.5602 -0.04 (-0.06, -0.02) 5.81e-05*
LPC(16:1)_[M+H]1+ / LPE(19:1)_[M+H]1+ 494.3245 -0.05 (-0.06, -0.03) 3.60e-11***
CE(16:1)_[M+NH4]1+ 640.6024 -0.05 (-0.07, -0.03) 2.46e-08**
FA(16:1)_[M-H]1- 253.2174 -0.07 (-0.10, -0.05) 5.80e-10***
Cer(42:11)_[M+OAc]1- 688.4947 -0.08 (-0.12, -0.04) 4.20e-05*
-.4 -.3 -.2 -.1 0 .1 .2 .3 .4
Beta (95% CI) for SCD (rs603424, chr10:102075479)

(u) *SGPP1*

Lipid name	Lipid *m/z*	Beta (95% CI)	*P*-value
SM(32:1)_[M+H]1+	675.5434	0.09 (0.07, 0.10)	8.21e-35***
SM(32:1)_[M-CH3]1-	659.5135	0.08 (0.06, 0.09)	4.31e-25***
PC-O(36:1)_[M+H]1+ / PE-O(39:1)_[M+H]1+ / PC-P(36:0)_[M+H]1+	774.6376	0.06 (0.03, 0.09)	1.92e-05*
SM(39:1)_[M+H]1+	773.6531	0.06 (0.04, 0.07)	3.41e-10***
SM(39:1)_[M-CH3]1-	757.623	0.05 (0.03, 0.07)	6.21e-08*
SM(38:0)_[M+H]1+	761.6532	0.05 (0.03, 0.07)	7.33e-08*
SM(33:1)_[M-CH3]1-	673.5291	0.05 (0.03, 0.06)	8.15e-09**
SM(33:0)_[M+OAc]1-	749.5815	0.04 (0.02, 0.06)	1.26e-04*
Cer(40:2)_[M-H]1-	618.5831	0.04 (0.01, 0.07)	5.67e-03
SM(38:1)_[M+H]1+	759.6372	0.03 (0.02, 0.05)	5.09e-07*
SM(37:1)_[M+H]1+	745.6216	0.03 (0.02, 0.05)	1.15e-04*
SM(38:1)_[M+CH3]1-	743.6075	0.03 (0.02, 0.05)	1.46e-04*
SM(33:1)_[M+OAc]1-	747.5659	0.03 (0.01, 0.05)	5.01e-04*
SM(41:2)_[M+H]1+	799.6685	0.03 (0.01, 0.04)	1.47e-03
SM(40:2)_[M-CH3]1-	769.6231	0.02 (0.01, 0.04)	4.37e-03
SM(34:2)_[M+H]1+	701.5593	0.02 (0.01, 0.03)	5.07e-03
PC(34:1)_[M+H]1+ / PE(37:1)_[M+H]1+ / PA(39:2)_[M+NH4]1+	760.5853	-0.02 (-0.03, -0.00)	7.81e-03
PE(36:0)_[M-H]1-	746.5704	-0.02 (-0.04, -0.01)	8.89e-03
PA(43:4)_[M-H]1-	793.5752	-0.02 (-0.04, -0.01)	8.07e-03
PG(35:2)_[M-H]1-	759.518	-0.03 (-0.05, -0.01)	1.11e-02

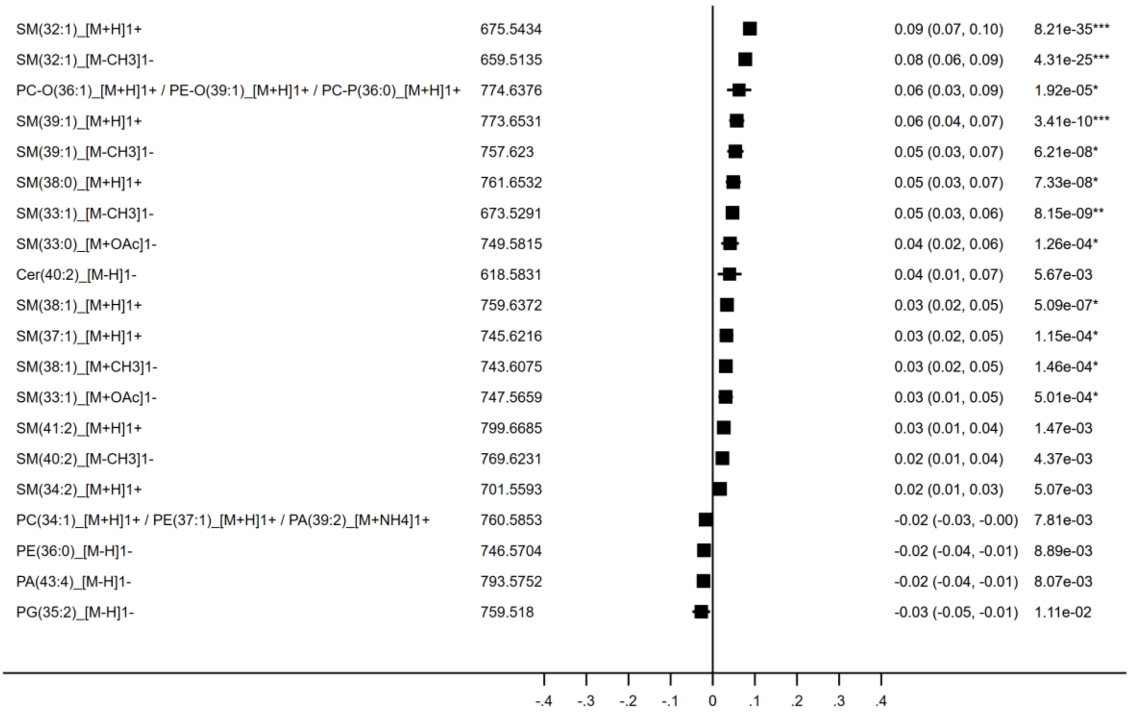

Beta (95% CI) for *SGPP1* (rs7157785, chr14:64235556)

(v) *SPTLC3*

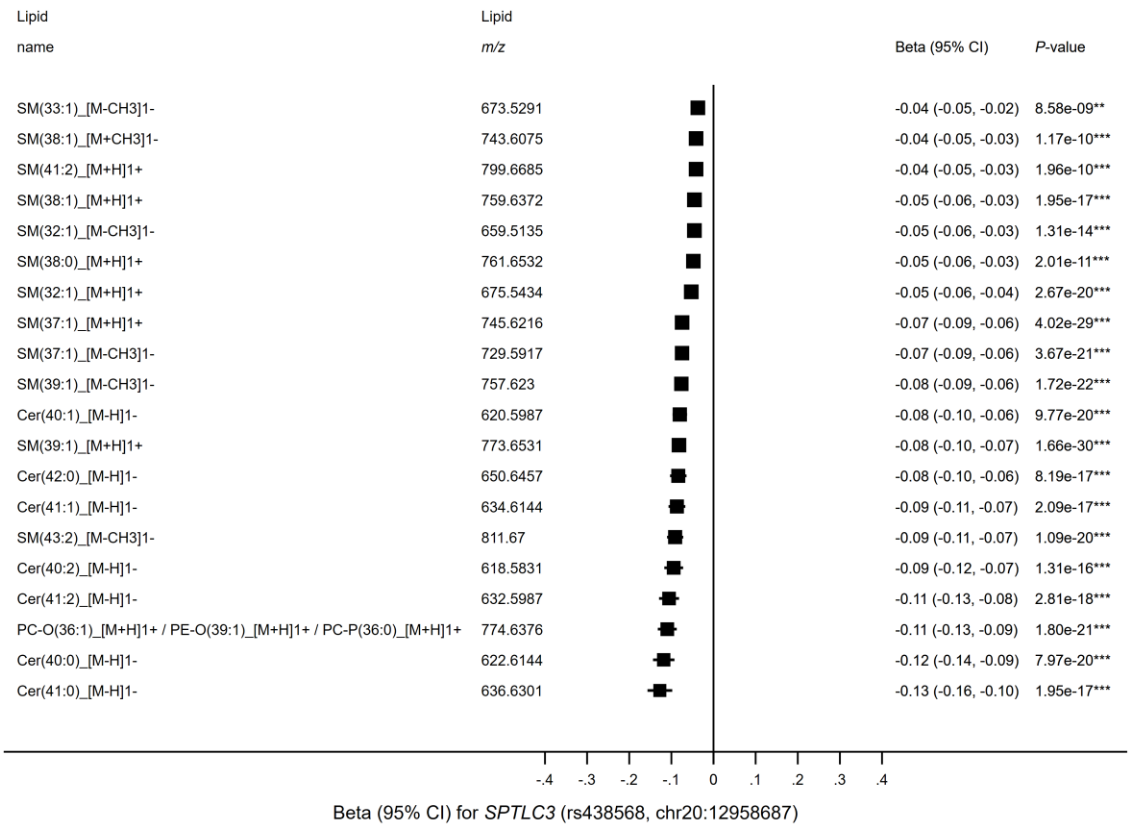

Lipid name
Lipid m/z
Beta (95% CI)
P-value
SM(33:1)_[M-CH3]1- 673.5291 -0.04 (-0.05, -0.02) 8.58e-09**
SM(38:1)_[M+CH3]1- 743.6075 -0.04 (-0.05, -0.03) 1.17e-10***
SM(41:2)_[M+H]1+ 799.6685 -0.04 (-0.05, -0.03) 1.96e-10***
SM(38:1)_[M+H]1+ 759.6372 -0.05 (-0.06, -0.03) 1.95e-17***
SM(32:1)_[M-CH3]1- 659.5135 -0.05 (-0.06, -0.03) 1.31e-14***
SM(38:0)_[M+H]1+ 761.6532 -0.05 (-0.06, -0.03) 2.01e-11***
SM(32:1)_[M+H]1+ 675.5434 -0.05 (-0.06, -0.04) 2.67e-20***
SM(37:1)_[M+H]1+ 745.6216 -0.07 (-0.09, -0.06) 4.02e-29***
SM(37:1)_[M-CH3]1- 729.5917 -0.07 (-0.09, -0.06) 3.67e-21***
SM(39:1)_[M-CH3]1- 757.623 -0.08 (-0.09, -0.06) 1.72e-22***
Cer(40:1)_[M-H]1- 620.5987 -0.08 (-0.10, -0.06) 9.77e-20***
SM(39:1)_[M+H]1+ 773.6531 -0.08 (-0.10, -0.07) 1.66e-30***
Cer(42:0)_[M-H]1- 650.6457 -0.08 (-0.10, -0.06) 8.19e-17***
Cer(41:1)_[M-H]1- 634.6144 -0.09 (-0.11, -0.07) 2.09e-17***
SM(43:2)_[M-CH3]1- 811.67 -0.09 (-0.11, -0.07) 1.09e-20***
Cer(40:2)_[M-H]1- 618.5831 -0.09 (-0.12, -0.07) 1.31e-16***
Cer(41:2)_[M-H]1- 632.5987 -0.11 (-0.13, -0.08) 2.81e-18***
PC-O(36:1)_[M+H]1+ / PE-O(39:1)_[M+H]1+ / PC-P(36:0)_[M+H]1+ 774.6376 -0.11 (-0.13, -0.09) 1.80e-21***
Cer(40:0)_[M-H]1- 622.6144 -0.12 (-0.14, -0.09) 7.97e-20***
Cer(41:0)_[M-H]1- 636.6301 -0.13 (-0.16, -0.10) 1.95e-17***
-.4 -.3 -.2 -.1 0 .1 .2 .3 .4
Beta (95% CI) for SPTLC3 (rs438568, chr20:12958687)

(w) *UGT8*

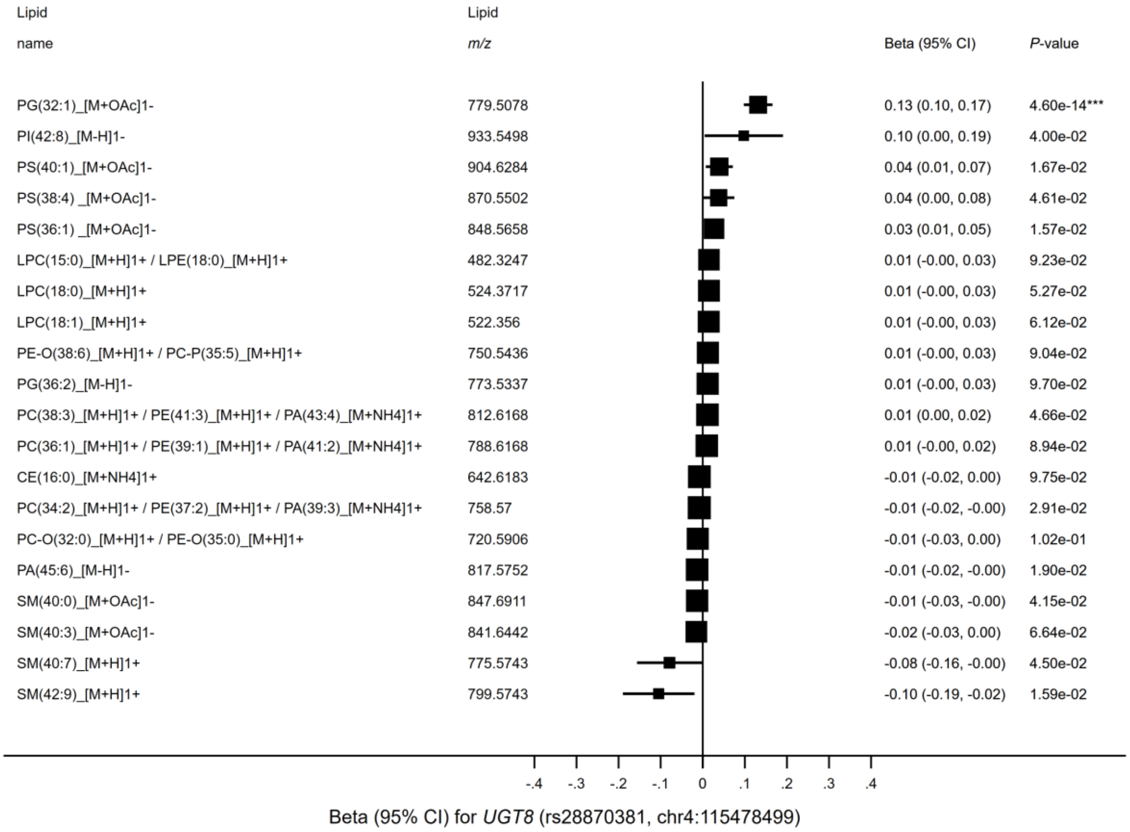
Lipid name
Lipid m/z
Beta (95% CI)
P-value
PG(32:1)_[M+OAc]1- 779.5078 0.13 (0.10, 0.17) 4.60e-14***
PI(42:8)_[M-H]1- 933.5498 0.10 (0.00, 0.19) 4.00e-02
PS(40:1)_[M+OAc]1- 904.6284 0.04 (0.01, 0.07) 1.67e-02
PS(38:4) _[M+OAc]1- 870.5502 0.04 (0.00, 0.08) 4.61e-02
PS(36:1) _[M+OAc]1- 848.5658 0.03 (0.01, 0.05) 1.57e-02
LPC(15:0)_[M+H]1+ / LPE(18:0)_[M+H]1+ 482.3247 0.01 (-0.00, 0.03) 9.23e-02
LPC(18:0)_[M+H]1+ 524.3717 0.01 (-0.00, 0.03) 5.27e-02
LPC(18:1)_[M+H]1+ 522.356 0.01 (-0.00, 0.03) 6.12e-02
PE-O(38:6)_[M+H]1+ / PC-P(35:5)_[M+H]1+ 750.5436 0.01 (-0.00, 0.03) 9.04e-02
PG(36:2)_[M-H]1- 773.5337 0.01 (-0.00, 0.03) 9.70e-02
PC(38:3)_[M+H]1+ / PE(41:3)_[M+H]1+ / PA(43:4)_[M+NH4]1+ 812.6168 0.01 (0.00, 0.02) 4.66e-02
PC(36:1)_[M+H]1+ / PE(39:1)_[M+H]1+ / PA(41:2)_[M+NH4]1+ 788.6168 0.01 (-0.00, 0.02) 8.94e-02
CE(16:0)_[M+NH4]1+ 642.6183 -0.01 (-0.02, 0.00) 9.75e-02
PC(34:2)_[M+H]1+ / PE(37:2)_[M+H]1+ / PA(39:3)_[M+NH4]1+ 758.57 -0.01 (-0.02, -0.00) 2.91e-02
PC-O(32:0)_[M+H]1+ / PE-O(35:0)_[M+H]1+ 720.5906 -0.01 (-0.03, 0.00) 1.02e-01
PA(45:6)_[M-H]1- 817.5752 -0.01 (-0.02, -0.00) 1.90e-02
SM(40:0)_[M+OAc]1- 847.6911 -0.01 (-0.03, -0.00) 4.15e-02
SM(40:3)_[M+OAc]1- 841.6442 -0.02 (-0.03, 0.00) 6.64e-02
SM(40:7)_[M+H]1+ 775.5743 -0.08 (-0.16, -0.00) 4.50e-02
SM(42:9)_[M+H]1+ 799.5743 -0.10 (-0.19, -0.02) 1.59e-02
-.4 -.3 -.2 -.1 0 .1 .2 .3 .4
Beta (95% CI) for UGT8 (rs28870381, chr4:115478499)

(x) *XBP1*

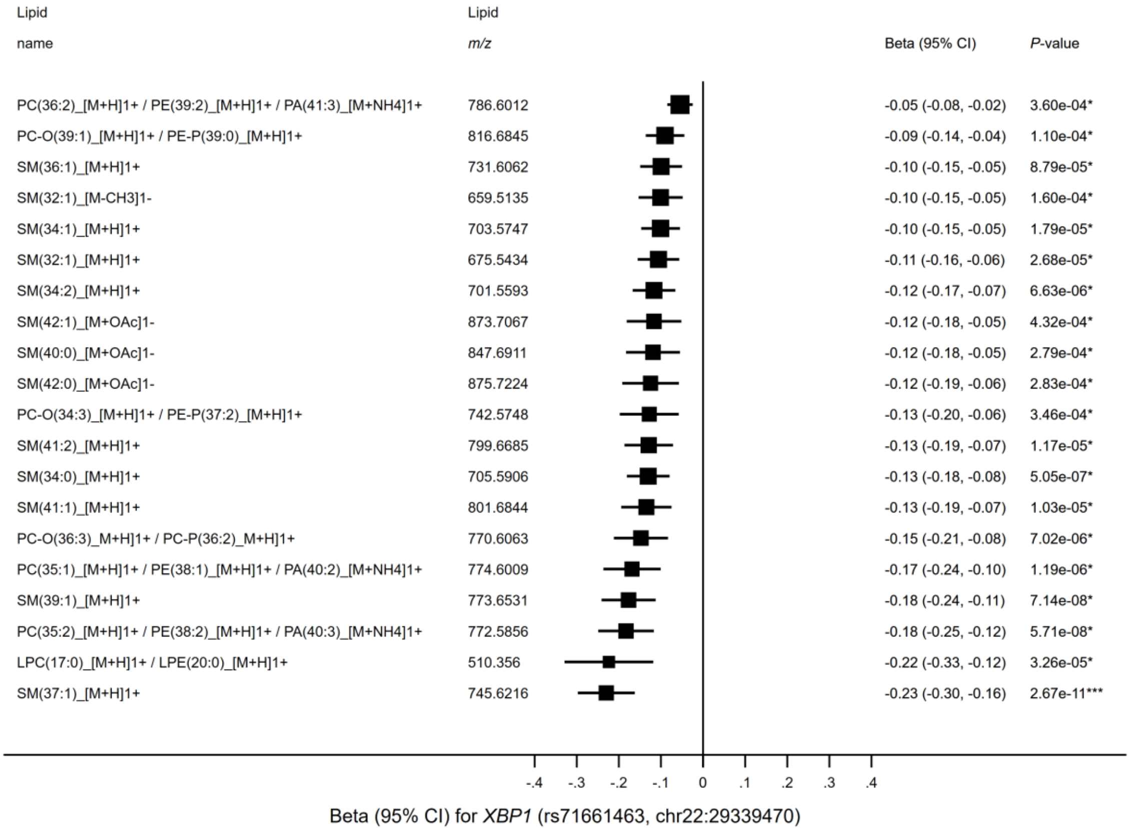

Forest plots showing the association of the top 20 most significantly associated lipids in PROMIS with the lead variant in each significant locus from the conditional analyses. **Note:** * = *P* < 0.001; ** = *P* < 5 x 10^{-8}; *** = *P* < 8.9 x 10^{-10}.

Supplementary Figure 8. Comparison of genetic associations with lipids pre- and post-adjustment for clinical lipid measures

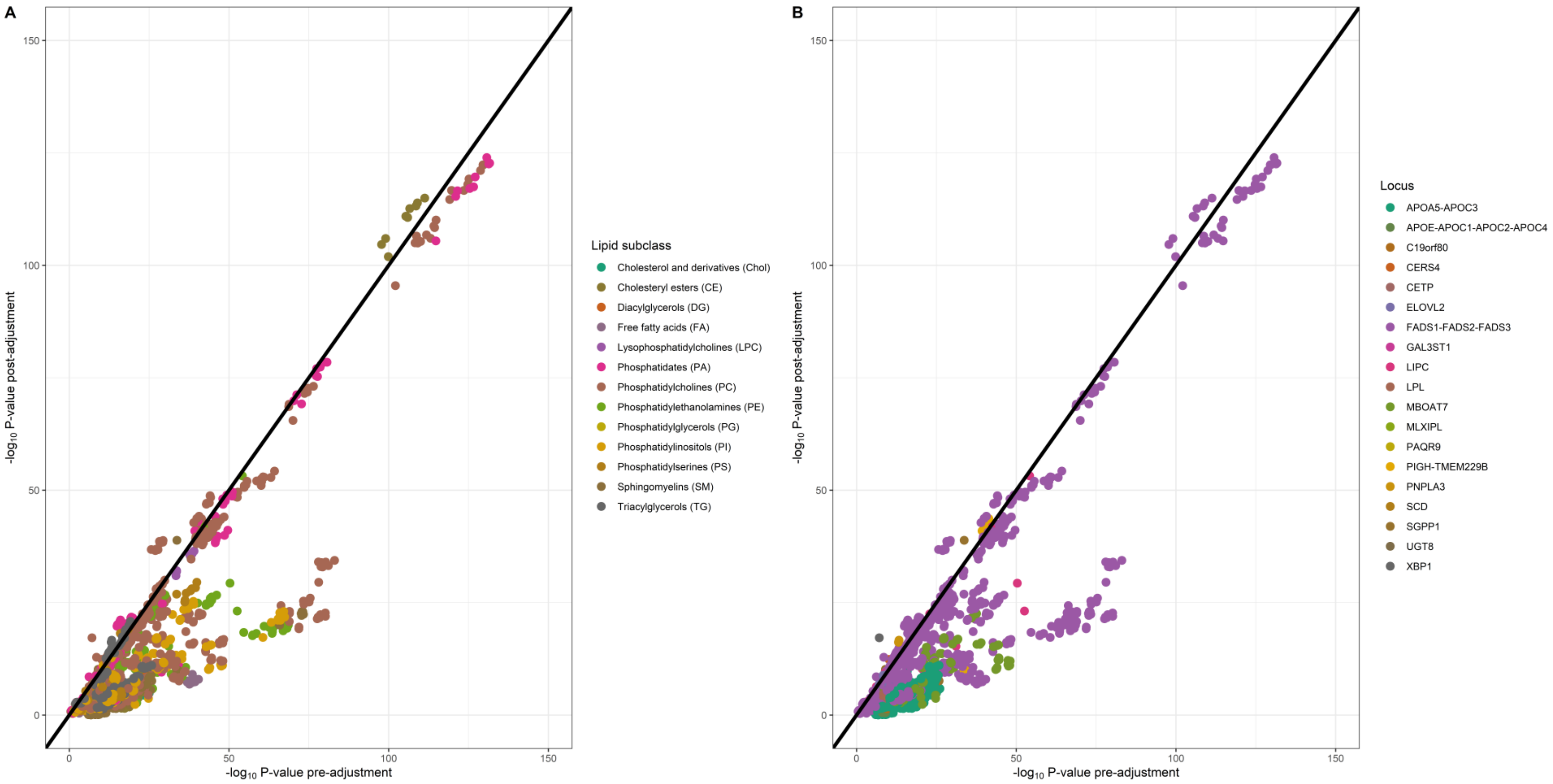

Comparison of $-\log_{10}$ *P*-values in PROMIS from conditional analyses (pre-adjustment) and after adjustment for several clinical lipid measures (total cholesterol, HDL cholesterol, and triglycerides). Association estimates are shown coloured by (a) lipid subclass and (b) genetic locus.
