# Supplementary figures and images for: Genome-wide analysis of blood lipid metabolites in over 5000 South Asians reveals biological insights at cardiometabolic disease loci
Source: BMC Med. 2021 Sep 10;19:232. doi: 10.1186/s12916-021-02087-1 (PMC8431908; doi:10.1186/s12916-021-02087-1)

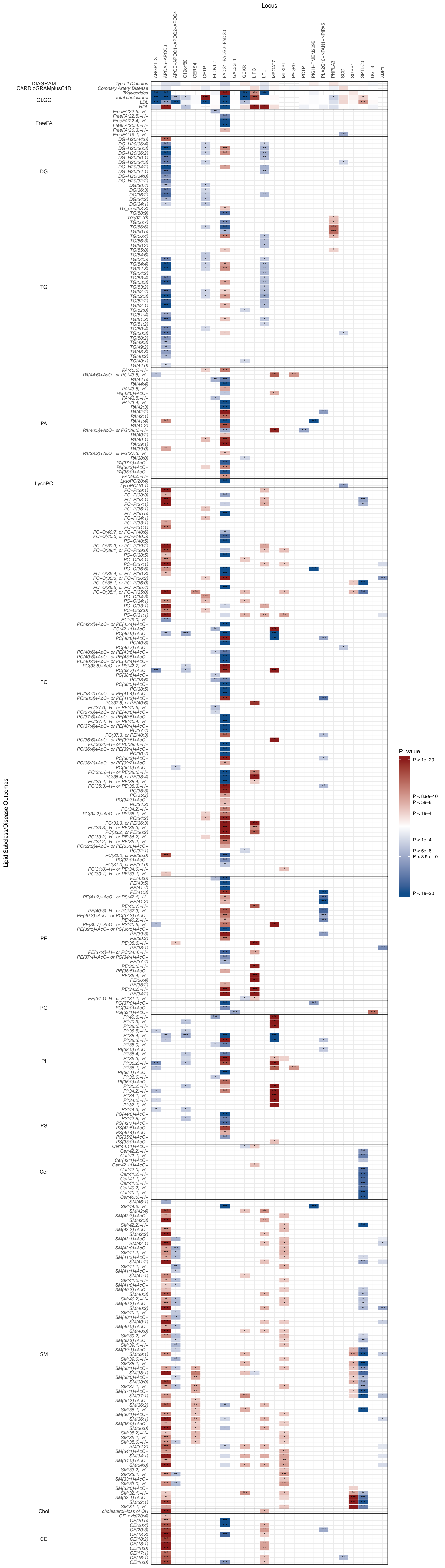

Supplement: Supplementary file 3 — Additional file 3. Supplementary Figure 1 (high resolution). [file 12916_2021_2087_MOESM3_ESM.pdf]
